# Thermoresponsive Poly(*N*,*N*′-dimethylacrylamide)-Based Diblock Copolymer Worm Gels via RAFT Solution Polymerization: Synthesis, Characterization, and Cell Biology Applications

**DOI:** 10.1021/acs.biomac.3c00635

**Published:** 2023-08-24

**Authors:** Damla Ulker, Thomas J. Neal, Aileen Crawford, Steven P. Armes

**Affiliations:** † Dainton Building, Department of Chemistry, University of Sheffield, Brook Hill, Sheffield, South Yorkshire S3 7HF, UK; ‡ Faculty of Pharmacy, Department of Pharmaceutical Basic Sciences, Near East University, Nicosia, Northern Cyprus TR-99138, Turkey; § School of Clinical Dentistry, University of Sheffield, Claremont Crescent, Sheffield, South Yorkshire S10 2TA, UK

## Abstract

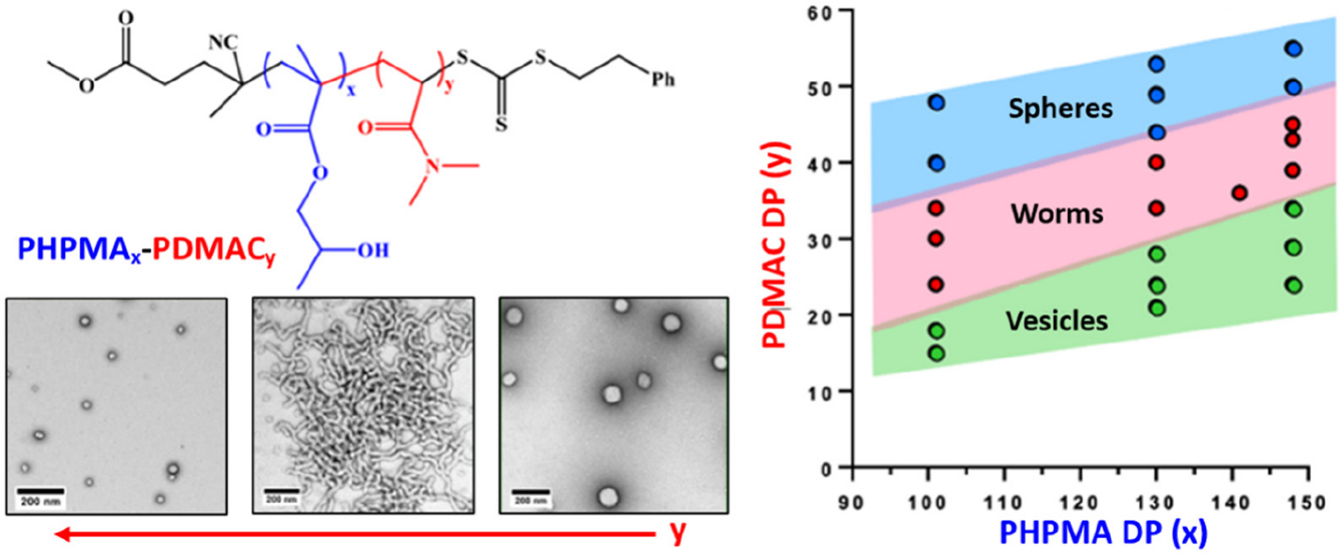

RAFT solution polymerization is used to polymerize 2-hydroxypropyl methacrylate (HPMA). The resulting PHPMA precursor is then chain-extended using *N*,*N*′-dimethylacrylamide (DMAC) to produce a series of thermoresponsive PHPMA-PDMAC diblock copolymers. Such amphiphilic copolymers can be directly dispersed in ice-cold water and self-assembled at 20 °C to form spheres, worms, or vesicles depending on their copolymer composition. Construction of a pseudo-phase diagram is required to identify the pure worm phase, which corresponds to a rather narrow range of PDMAC DPs. Such worms form soft, free-standing gels in aqueous solution at around ambient temperature. Rheology studies confirm the thermoresponsive nature of such worms, which undergo a reversible worm-to-sphere on cooling below ambient temperature. This morphological transition leads to *in situ* degelation, and variable temperature ^1^H NMR studies indicate a higher degree of (partial) hydration for the weakly hydrophobic PHPMA chains at lower temperatures. The trithiocarbonate end-group located at the end of each PDMAC chain can be removed by treatment with excess hydrazine. The resulting terminal secondary thiol group can form disulfide bonds via coupling, which produces PHPMA-PDMAC-PHPMA triblock copolymer chains. Alternatively, this reactive thiol group can be used for conjugation reactions. A PHPMA_141_-PDMAC_36_ worm gel was used to store human mesenchymal stem cells (MSCs) for up to three weeks at 37 °C. MSCs retrieved from this gel subsequently underwent proliferation and maintained their ability to differentiate into osteoblastic cells.

## Introduction

Reversible addition-fragmentation chain transfer (RAFT) polymerization is an important example of a pseudo-living polymerization, not least because its radical-based chemistry is tolerant of a wide range of functional vinyl monomers.^1−5^ Following pioneering studies by Hawkett and co-workers in 2002, RAFT polymerization has been widely used for the synthesis of well-defined block copolymer nano-objects via polymerization-induced self-assembly (PISA).^6^ Typically, PISA involves growing an insoluble block from one end of a soluble precursor block in a suitable solvent. This leads to the diblock copolymer chains undergoing *in situ* self-assembly to form sterically stabilized diblock copolymer nano-objects.^3,7,8^ PISA offers faster rates of reaction and higher final monomer conversions than the equivalent solution polymerization because the polymerization occurs within monomer-swollen particles, which leads to a relatively high local monomer concentration.^9^ Moreover, the desired nanoparticles are obtained directly in the form of a concentrated colloidal dispersion and typically require no further processing.^10^

Over the past decade, we have reported a series of thermoresponsive diblock copolymer worm gels based on poly(2-hydroxypropyl methacrylate) (PHPMA) using aqueous PISA formulations.^11−36^ Initially, the water-soluble steric stabilizer block comprised dihydroxyl-functional poly(glycerol monomethacrylate (PGMA),^14,19,22,36^ with subsequent examples including monohydroxy-functional poly(2-hydroxypropyl methacrylamide) (PHPMAC),^30^ non-ionic poly(ethylene glycol) (PEG),^18,31^ zwitterionic poly(2-(methacryloyloxy)ethyl phosphorylcholine) (PMPC),^17,28,32^ and anionic poly(methacrylic acid) (PMAA).^33−35^ In each case, pure worms occupy relatively narrow phase space. For a fixed steric stabilizer DP, the mean PHPMA DP can typically vary by just 10–20 repeat units, with mixed phases being obtained either side of this rather limited range. In practice, this means that the painstaking construction of a suitable pseudo-phase diagram is required to ensure reproducible targeting of the worm morphology.^7,8,11,17,18,25,27−29,31,33^ Moreover, the absolute degree of polymerization (DP) of the PHPMA block also requires optimization to ensure thermoreversible behavior: if it is too long, then it remains permanently hydrophobic and its desirable thermoresponsive character is lost.^12,25,28,36^ In semiconcentrated aqueous solution, multiple physical contacts between neighboring worms lead to the formation of soft, free-standing transparent gels at ambient temperature.^37^ Such gels comprise an open, highly porous network, which enables the rapid diffusion of small molecule nutrients and waste products into and out of the gel. On cooling to 4–5 °C, a worm-to-sphere transition occurs, which leads to *in situ* degelation.^38^ This change in morphology is driven by a subtle variation in the degree of (partial) hydration of the PHPMA block; this is sufficient to lower the fractional packing parameter, which favors the formation of spheres.^34^ Essentially, the same worm gel is reformed on returning to 20–25 °C, which enables facile sterilization via cold ultrafiltration.^38^ In the case of the PGMA-PHPMA worm gels, their excellent biocompatibility has enabled their use as a convenient 3D matrix for long-term cell culture studies.^39^ In contrast, if embryonic human stem cells are immersed into such worm gels in the form of colonies, then cell stasis is observed—the stem cells become dormant and can survive at 37 °C in this form for up to two weeks without any loss of pluripotency.^40^ This suggests that human stem cells could be stored within such worm gels and shipped from cell manufacturing facility to the clinic without recourse to cryopreservation. This is a potentially important finding because only a minority of stem cells survive the thawing process after being cryogenically frozen.^41^ Subsequent cell biology experiments conducted with PGMA-PHPMA and PEG-PHPMA worm gels of comparable softness suggest that the hydroxyl-rich PGMA block is essential for stasis induction: only cell proliferation was observed for stem cell colonies immersed within PEG-PHPMA worm gels.^39^

In the present study, we examine the synthesis of thermoresponsive PHPMA-based worm gels using poly(*N*,*N*′-dimethylacrylamide) (PDMAC) as the hydrophilic steric stabilizer block. Our motivation was to examine whether such non-ionic PDMAC chains promote stasis induction (like PGMA) or cell proliferation (like PEG chains). Unfortunately, PDMAC-PHPMA diblock copolymers cannot be prepared using a conventional aqueous PISA formulation owing to poor cross-initiation efficiency when attempting to use the acrylamide-based precursor for the polymerization of a methacrylic monomer such as HPMA.^42,43^ Moreover, a new reverse sequence PISA formulation also proved to be ineffective when targeting PHPMA-PDMAC diblock copolymers.^44^ In view of these problems, we decided to prepare such diblock copolymers via RAFT solution polymerization in ethanol. This alternative approach should be feasible because PGMA-PHPMA worm gels can be reconstituted from dry powder via direct dissolution in cold water followed by warming to ambient temperature.^45^ This protocol ensures molecular dissolution of the copolymer chains prior to their *in situ* self-assembly to form worms without recourse to any organic cosolvent. Similar self-assembly behavior was anticipated for the new PHPMA-PDMAC worms targeted herein. Furthermore, PHPMA-PDMAC worm gels prepared via this new strategy were expected to be sufficiently biocompatible for cell biology applications. More specifically, we investigated the osteogenic differentiation of human bone marrow mesenchymal stem cells (hBM-MSCs) via alkaline phosphatase activity after encapsulation within such worm gels for up to two weeks at 37 °C.

## Experimental Section

### Materials

2-Hydroxypropyl methacrylate (HPMA) was kindly donated by GEO Specialty Chemicals (Hythe, UK), and *N*,*N-*dimethylacrylamide (DMAC) was purchased from Sigma-Aldrich (Dorset, UK). These two monomers were passed in turn through a basic alumina column to remove inhibitors. Azobisisobutyronitrile (AIBN) was purchased from Molekula GmbH (Germany). Ethanol (99.8%) and diethyl ether (≥99.8%) were purchased from Fisher Scientific (Loughborough, UK) and Sigma-Aldrich (Dorset, UK), respectively. Methyl 4-cyano-4-(2-phenylethanesulfanylthiocarbonyl)sulfanyl pentanoate (MePETTC) was prepared in-house as reported previously.^19^ Propylamine and dithiothreitol (DTT) were purchased from Sigma-Aldrich (Dorset, UK). Dialysis tubing (molecular weight cutoff = 10,000) was purchased from Thermo Fisher Scientific (Rockford, USA). Phosphate buffer saline tablet was purchased from Oxoid (Hampshire, UK) for PBS solution. Deionized water was used for all experiments. CD_3_OD and Cl_2_CD_2_ were purchased from Cambridge Isotherm Laboratories (Tewksbury, UK).

Primary hBM-MSCs were purchased from PromoCell (Heidelberg, Germany). Dulbecco’s Modified Eagle Medium (DMEM, “low-glucose” formula comprising 1 g dm^–3^ glucose), l-glutamine, and nonessential amino acids were purchased from Sigma-Aldrich (Dorset, UK). MSC-qualified foetal bovine serum (FBS) was purchased from ThermoFisher Scientific UK, (Paisley, UK); stabilized stock solution containing 50,000 U penicillin and 50,000 μg streptomycin per mL was purchased from Sigma-Aldrich (Dorset, UK). 0.25% trypsin/0.2% EDTA solution, phosphate buffered saline (PBS) tablets (for cell studies), and PrestoBlue cell viability reagent and ProLong Diamond anti-fade mountant were purchased from ThermoFisher Scientific UK (Paisley, UK). The recombinant rabbit anti-human Ki-67 antibody [SP6] (ab16667) and goat anti-rabbit IgG H&L (Alexa Fluor 488) (ab 150081) were purchased from Abcam (Cambridge, UK). Dexamethasone, β-glycerophosphate, ascorbic acid, *p*-nitrophenol phosphate, glycine, MgCl_2_, fibronectin, 10% neutral buffered formalin solution, 4% buffered formaldehyde solution pH 6.9, and Nunc Lab-Tek 4-well Permanox chamber slides were purchased from Sigma-Aldrich (Dorset, UK). Cell + tissue culture flasks and cell + 24-well plates were purchased from Sarstedt UK (Leicester, UK), and 0.22 μm filters were purchased from ThermoFisher Scientific UK, (Leicestershire, UK).

## Methodology

### Synthesis of the PHPMA_*x*_ Precursor by RAFT Solution Polymerization of HPMA in Ethanol

The RAFT solution polymerization of HPMA was conducted in ethanol at 70 °C. A typical synthesis was conducted as follows. HPMA (40.0 g, 279 mmol), MePETTC (0.885 g, 2.49 mmol; target DP = 140), and AIBN (0.082 g, 0.500 mmol; MePETTC/AIBN molar ratio = 5.0) were weighed into a 250 mL round-bottomed flask. Anhydrous ethanol (173 mL) was added to the flask to produce a 30% w/w solution, which was placed in an ice bath and purged under nitrogen for 30 min at 0 °C. The sealed flask was then immersed in an oil bath set at 70 °C to initiate the RAFT solution polymerization of HPMA. After 6 h, the polymerization was quenched by exposing the reaction solution to air while cooling the flask to room temperature. ^1^H NMR spectroscopy studies indicated an HPMA conversion of 70%. The reaction solution was added to a 9-fold excess of diethyl ether to isolate the rude PHPMA via precipitation, which removed unreacted monomer, initiator residues, and trace amounts of RAFT agents. The precipitate was isolated via filtration, and this protocol was repeated three times. The purified copolymer was dried overnight in a vacuum oven at 37 °C. ^1^H NMR analysis indicated a mean DP of 101 for this PHPMA precursor as estimated by monomer conversion (the integrated vinyl monomer signals at 6.05–6.25 ppm assigned to the two backbone protons at 1.7–2.4 ppm). DMF GPC analysis (see below for further details) indicated an *M*_n_ of 18,700 g mol^–1^ and an *M*_w_/*M*_n_ of 1.18. Systematically varying the HPMA/Me-PETTC molar ratio using the same reaction conditions afforded a series of PHPMA precursors with mean DPs ranging from 101 to 148. Each copolymer was analyzed by ^1^H NMR spectroscopy and DMF GPC (see Table 1). After polymerization, the PHPMA precursors were purified using three times precipitation in 10-fold excess ethyl ether.

**Table 1:** Summary of the Molecular Weight Data for the Three PHPMA Precursors and Corresponding PHPMA-PDMAC Diblock Copolymers Used in This Study

copolymer compositions	*M*_n_^a^ (kg mol^–1^)	*M*_w_/*M*_n_^a^	*M*_n_^b^ (kg mol^–1^)
PHPMA_101_	18.74	1.18	14.81
PHPMA_101_-PDMAC_15_	20.14	1.21	16.26
PHPMA_101_-PDMAC_18_	20.87	1.21	16.59
PHPMA_101_-PDMAC_24_	21.85	1.19	17.22
PHPMA_101_-PDMAC_30_	22.20	1.19	17.75
PHPMA_101_-PDMAC_34_	22.78	1.19	18.17
PHPMA_101_-PDMAC_39_	22.84	1.23	18.80
PHPMA_101_-PDMAC_50_	23.48	1.22	19.60
PHPMA_130_	21.55	1.15	19.04
PHPMA_130_-PDMAC_21_	23.22	1.19	20.08
PHPMA_130_-PDMAC_24_	25.86	1.16	21.43
PHPMA_130_-PDMAC_28_	25.70	1.15	21.84
PHPMA_130_-PDMAC_34_	26.65	1.15	22.41
PHPMA_130_-PDMAC_40_	26.84	1.17	22.98
PHPMA_130_-PDMAC_44_	26.87	1.16	23.38
PHPMA_130_-PDMAC_49_	27.01	1.16	23.92
PHPMA_130_-PDMAC_53_	27.30	1.16	24.27
PHPMA_141_	22.54	1.16	20.52
PHPMA_141_-PDMA_36_^c^	29.68	1.16	24.12
PHPMA_148_	22.78	1.15	21.57
PHPMA_148_-PDMAC_24_	25.95	1.19	23.94
PHPMA_148_-PDMAC_29_	26.12	1.19	24.44
PHPMA_148_-PDMAC_34_	26.57	1.19	24.94
PHPMA_148_-PDMAC_39_	27.01	1.20	25.47
PHPMA_148_-PDMAC_43_	27.43	1.18	25.87
PHPMA_148_-PDMAC_45_	27.93	1.18	26.21
PHPMA_148_-PDMAC_50_	28.06	1.17	26.52
PHPMA_148_-PDMAC_56_	28.35	1.14	25.17

a Molecular weight data determined by DMF GPC (refractive index detector, vs PMMA standards).

b *M*_n_ determined by end-group analysis using ^1^H NMR spectroscopy.

c The PHPMA_141_-PDMA_36_ diblock copolymer was used for the biological applications.

### Synthesis of PHPMA_*x*_-PDMAC_*y*_ Diblock Copolymers via RAFT Solution Polymerization of DMAC in Ethanol

A series of PHPMA-PDMAC diblock copolymers were prepared via RAFT solution polymerization of DMAC in ethanol. The following synthesis is representative. A PHPMA_101_ precursor (1.33 g, 0.105 mmol), DMAC monomer (0.15 g, 1.51 mmol; target DP = 20), and AIBN (3.10 mg, 19 μmol; PHPMA_101_/AIBN molar ratio = 5.0) were added to a 25 mL round-bottomed flask, followed by addition of ethanol (4.7 mL) to produce a 40% w/w solution. The flask was immersed in an ice bath, and the cold reaction solution was purged with nitrogen gas for 30 min to remove oxygen. Then, the sealed flask was immersed in an oil bath set at 70 °C to initiate the RAFT solution polymerization. After 6 h, the polymerization was quenched by exposing the reaction mixture to air while cooling the flask to room temperature. The DMAC conversion was calculated to be 90% from the attenuation of the monomer vinyl signals at 6.1 ppm. The mean PDMAC DP was calculated by comparing the integrated the vinyl protons of DMAC monomer at 6.14–6.30 (2H, −CH_2_) with the methyl protons assigned to the DMAC repeat units at 2.87–3.26 ppm (6H, N(CH_3_)_2_). DMF GPC analysis indicated that such diblock copolymers exhibited relatively narrow molecular weight distributions (*M*_w_/*M*_n_ ≤ 1.20). The mass of DMAC monomer used for the synthesis of PHPMA-PDMAC was kept constant for all reactions, and the target PDMAC DP was varied from 15 to 50 by adjusting the mass of the PHPMA precursor.

### Copolymer Purification and Aqueous Self-Assembly Protocol

After the RAFT solution polymerization of DMAC in ethanol, the copolymer concentration was diluted to 20% w/w. Removal of small molecule impurities was achieved via dialysis against ethanol for three days, followed by dialysis against water for seven days. The purified copolymer was then freeze-dried overnight. The resulting yellow copolymer was molecularly dissolved in ice-cold water (or aqueous PBS) and then allowed to warm up to ambient temperature to induce *in situ* self-assembly. A copolymer concentration of 10% w/w was used to produce worm gels in purely aqueous media, whereas a copolymer concentration of 5% w/w was employed to produce worm gels in aqueous PBS.

### Preparation of PHPMA-PDMAC Worm Gels for Stem Cell Studies

It is well-known that organosulfur-based RAFT end-groups can influence the biocompatibility of a given copolymer.^46^ Moreover, their *in situ* hydrolytic degradation generates malodorous, potentially cytotoxic sulfur-based compounds.^47^ Thus, RAFT end-group removal is likely to enhance copolymer biocompatibility.^40,48^ This was achieved by treating an aqueous dispersion (10 w/w) of a PHPMA_141_-PDMAC_36_ diblock copolymer (1.86 g, 70 μmol) with a 20-fold excess of *n*-propylamine for 24 h at 20 °C. According to the literature, this protocol produces thiol-capped copolymer chains.^49,50^ UV GPC (λ = 305 nm) was used to determine the extent of trithiocarbonate end-group removal achieved under such conditions.

## Characterization

### ^1^H NMR Spectroscopy

Spectra were recorded at 20 °C using a 400 MHz Bruker Avance-400 spectrometer in CD_2_Cl_2_, CD_3_OD or D_2_O. Typically, 64 scans were averaged per spectrum, and chemical shifts are reported in ppm (δ). Variable temperature ^1^H NMR studies were performed in D_2_O from 5 to 50 °C to assess the variable degree of hydration of the PHPMA block.

### Gel Permeation Chromatography (GPC)

Each PHPMA precursor and the corresponding series of PHPMA-PDMAC diblock copolymers were analyzed using an Agilent 1260 Infinity GPC system operating at 60 °C and equipped with both refractive index and UV–vis detectors. This setup comprised two Polymer Laboratories PL gel 5 μm Mixed C columns and HPLC-grade DMF eluent containing 10 mM LiBr; the flow rate was 1.0 mL min^–1^. The refractive index detector was used to calculate molecular weight data via calibration using 10 near-monodisperse poly(methyl methacrylate) standards. UV chromatograms were recorded simultaneously at a fixed wavelength of 309 nm, which corresponds to the absorption maximum for the trithiocarbonate-based RAFT end-groups.

### Dynamic Light Scattering (DLS)

The *z*-average diameter was determined for aqueous copolymer dispersions at various temperatures using a Malvern Zetasizer NanoZS instrument at a fixed scattering angle of 173°. Copolymer dispersions were diluted to 0.1% w/w using either deionized water or aqueous PBS. Data were averaged over three consecutive measurements (using 10 subruns per run). The hydrodynamic diameter was calculated using the Stokes-Einstein equation, which assumes perfectly monodisperse, noninteracting spheres. Clearly, this is not the case for block copolymer worms.^51^ In this case, DLS reports a spherical-average diameter that corresponds to neither the mean worm length nor the mean worm width. Nevertheless, DLS can still indicate the characteristic temperature required to induce the worm-to-sphere transition. Temperature sweeps were conducted at 2 °C intervals with 5 min being allowed for thermal equilibrium at each temperature. In addition, apparent zeta potentials were determined via the Henry equation using the Smoluchowski approximation. In this case, 0.1% w/w aqueous dispersions were prepared using 1 mM KCl, with the dispersion pH being adjusted using either 0.1 or 1 M HCl as required.

### Transmission Electron Microscopy (TEM)

Copper/palladium TEM grids (Agar Scientific, UK) were surface-coated in-house to produce a thin film of amorphous carbon. Then, the grids were plasma glow-discharged for 30 s to create a hydrophilic surface. An 8 μL droplet of a 0.03% w/w aqueous copolymer dispersion (prepared by dilution of a 10% w/w copolymer gel or dispersion at 37, 20, or 4 °C, respectively) was placed on a surface-treated grid for 1 min, and then excess dispersion was removed using filter paper. Then, an 8 μL droplet of a 0.75% w/v aqueous solution of uranyl formate (negative stain) was added to the sample-loaded grid for 30 s, and excess stain was removed using filter paper. Each grid was dried using a vacuum hose, and imaging was performed on a Phillips CM100 instrument at 100 kV equipped with a Gatan 1 k CCD camera.

### Rheology

Rheology measurements were conducted using an MCR502 Anton Paar rheometer equipped with a variable temperature Peltier plate. A core-and-plate geometry (25 mm 1° aluminum cone) was used for all experiments. The storage (*G*′) and loss (*G*″) moduli for aqueous dispersions of PHPMA_148_-PDMAC_39_ worm gels were determined at various copolymer concentrations to determine the critical gelation concentration (CGC) at both 20 and 37 °C. In addition, rheology was also used to examine the thermoreversible behavior of such worm gels between 4 and 37 °C. Temperature sweeps were conducted at 2 °C intervals with 3 min being allowed for thermal equilibrium at each temperature. Typically, measurements were repeated three times at each temperature.

### Biological Experiments

#### Stem Cell Culture

Cell + culture flasks and culture plates and MSC-qualified foetal bovine serum (FBS) were used for all MSC culture and experiments. Primary human bone marrow mesenchymal stem cells (MSCs) were cultured in DMEM containing 2 mM l-glutamine, 1% nonessential amino acids, and 10% MSC-qualified FBS and 50 U/mL penicillin and 50 μg/mL streptomycin (MSC culture medium, MSC-CM). The culture flasks were incubated at 37 °C in a humidified atmosphere comprising 95% air plus 5% CO_2_. MSCs were passaged using 0.25% trypsin/0.02% EDTA to release the cells from the culture surface. These cells were removed and pelleted by centrifugation at 1000 rpm (196 g). The resulting pellets were resuspended in MSC-CM, and the MSCs were plated in fresh culture flasks at 1.3 × 10^4^ cells/cm^2^ and incubated as described above.^52,53^ MSC cells at passage 5–6 were used for all experiments.

#### MSC Encapsulation within Worm Gels

Aqueous copolymer dispersions comprising either PGMA_55_-PHPMA_135_ (6% w/w) or PHPMA_141_-PDMAC_36_ (4% w/w) were used for MSC encapsulation. PGMA_55_-PHPMA_135_ was used as a positive control. Worm gels were prepared by dissolving each copolymer in turn, in serum-free MSC-CM at 4 °C. The resulting cold solutions were sterilized by passing through 0.22 μm filters. Sterile FBS was added to these copolymer solutions to ensure a final concentration of 6% w/w PGMA_55_-PHPMA_135_ containing 10% FBS or 4% w/w PHPMA_141_-PDMAC_36_ containing 10% FBS. Then, cultured MSCs were harvested from the tissue culture flasks using 0.25% trypsin/0.02% EDTA and pelleted by centrifugation at 1000 rpm (196 g) for 5 min. The pelleted cells were resuspended in PBS and centrifuged for a second time. The resulting cell pellet was resuspended in MSC-CM, and the cell number was determined using a hemocytometer. The required number of cells for encapsulation was calculated for each copolymer solution to give a cell density of 10^6^ MSCs per mL of copolymer solution.

The cell suspensions containing the required number of MSCs for each copolymer were pelletized by centrifugation (5 min, 1000 rpm, 196 g). Aqueous supernatants were removed, and cell pellets were resuspended in the copolymer solution at 4 °C to yield a cell density of 1 × 10^6^ MSCs per mL for each copolymer. Gels were obtained by pipetting 150 μL aliquots (150,000 MSCs) of the copolymer plus cell suspensions into 24-well culture plates coated with 2% agarose and kept at a 45° angle until the gels began to form. The culture plates were then transferred to a 37 °C incubator and incubated at a 45° angle in a humidified atmosphere comprising 95% air plus 5% CO_2._ When the gels had set, 1 mL of prewarmed (37 °C) MSC-CM was added to each well and the culture plates were returned to the incubator in the usual horizontal positions. The gel-encapsulated MSC cells were then cultured in MSC-CM at 37 °C for up to three weeks with culture media changes conducted twice per week.

#### Cell Viability Assays

PrestoBlue was used to assess the viability of MSCs encapsulated within worm gels. End-point analyses were performed as previously described.^52,54^ PrestoBlue is a nontoxic, resazurin dye, which, in viable cells, is reduced to its fluorescent form (resorufin) by the NADH, FADH, NADPH, and cytochromes were produced by the cellular metabolism of living cells.^55^ Hence, determination of the level of resorufin is a measurement of the metabolic activity of the living cells. MSCs encapsulated within 150 μL of either 6% w/w PGMA_55_-PHPMA_135_ or 4% w/w PHPMA_141_-PDMAC_36_ worm gels were incubated with MSC-CM (450 μL) containing PrestoBlue dye (50 μL) for 1 h at 37 °C in a humidified atmosphere comprising 95% air plus 5% CO_2_. To assess the extent of any noncellular reduction of the dye, a reagent blank was included in which PrestoBlue (50 μL) was added to 450 μL of cell culture medium alone (i.e., in the absence of any cells) and incubated using the same protocol employed for the cell culture. After incubation, aliquots of the culture medium (200 μL) were transferred to a 96-well plate and the fluorescence owing to the formation of resorufin was determined at an excitation wavelength of 560 nm and an emission wavelength of 590 nm using a Tecan plate reader (Tecan Infinite 200, Tecan, Männedorf, Switzerland). Cell activity was calculated by subtracting the fluorescence value for the reagent blank from that of gels containing the encapsulated cells.

#### MSC Release from Worm Gels

Culture plates containing the cell-loaded worm gels were placed in an ice bath to induce degelation and hence release the MSCs. The resulting cold copolymer solution containing the free MSC cells was diluted with PBS and centrifuged at 200*g* for 5 min. Aqueous supernatants were removed and the cell pellets were washed with MSC-CM prior to further centrifugation (200*g* for 5 min). Finally, the pellets were resuspended in MSC-CM and the cells were pipetted into 24-well plates (with each well containing MSC cells released from a 150 μL aliquot of worm gel).

#### DNA Quantification

MSCs were released from worm gels as described above and then transferred to Eppendorf tubes and centrifuged at 200*g* for 5 min to form cell pellets. The cell pellets were resuspended in PBS, and each suspension was centrifuged once more to form a cell pellet. Each aqueous supernatant was removed, and 500 μL of 10 mM TrisHCl plus 1 mM EDTA (pH 7.4) was added to each Eppendorf tube followed by cell resuspension. The resulting cell suspensions were immediately transferred to a −80 °C freezer, and after freezing, they were thawed at room temperature. Cell suspensions were subjected to three of these freeze–thaw cycles to induce membrane rupture, and the DNA content of these MSC cells was quantified using a Picogreen dye assay^56^ according to the manufacturer’s instructions provided with this reagent. DNA quantification data were analyzed using a calibration curve constructed using calf thymus DNA.

#### Live-Dead Staining

MSCs were released from the worm gels as described above, and the cell pellet resuspended in protein-free MSC-CM containing 1 μM CMFDA (5-chloromethylfluotrescein diacetate) and 5 μM propidium iodide. The MSCs were incubated at 37 °C for 45 min, after which the cells were pelleted by centrifugation (200*g* for 5 min) and the pellet was washed three times with MSC-CM. On the final MSC resuspension, the cells were transferred to a 24-well plate and fixed with 10% buffered formalin, and the cells were examined with a Leica Thunder microscope. Live cells show green fluorescence dude to uptake of the CMFDA, and dead cells show nuclei stained with the propidium iodide, which are seen as a red fluorescence.

#### Osteogenic Differentiation

Osteogenesis assays were performed to assess whether the MSCs released from the worm gels were still able to undergo differentiation after their storage within either 4% w/w PHPMA_141_-PDMAC_36_ or 6% w/w PGMA_55_-PHPMA_135_ (positive control) worm gels. MSCs were released from the worm gels as described above and cultured to 90% confluence. Then, the cells were passaged (1:3) into fresh 24-well plates and cultured to confluence. The resulting MSCs were incubated for three weeks using either a control medium (DMEM plus 2% MSC-qualified FBS) or a classical osteogenic medium^57^ comprising DMEM, 4% MSC-qualified FBS, 10^–8^ M dexamethasone, 50 mg dm^–3^l-ascorbic acid, and 10 mM β-glycerophosphate, with media changes twice per week. After three weeks, the culture media were removed and the MSC monolayers were washed with PBS. After removal of this buffer solution, ice-cold methanol (400 μL) was added to each well and the culture plates were incubated for 10 min at −20 °C to fix the cell monolayers. The methanol was then removed, and the monolayers were washed with distilled water. After washing, the culture plates were drained, sealed with Parafilm, and stored at −20 °C for determination of alkaline phosphatase activity.

#### Alkaline Phosphatase Activity

Alkaline phosphatase is a well-known marker for osteogenic cells.^58,59^ Confluent cell monolayers of worm gel retrieved MSCs were incubated at 37 °C in the presence of an aqueous solution comprising 5 mM *p*-nitrophenol phosphate, 0.2 M glycine-NaOH buffer (pH 9.4), and 1 mM MgCl_2_ (300 μL per well). After incubation, a 200 μL aliquot of this buffer solution was removed from each well and transferred to a 96-well. NaOH (50 μL, 0.5 M) was added per well to quench the alkaline phosphatase reaction by raising the pH to pH 14, and the optical density (OD) of the samples was determined immediately using a Tecan plate reader at a wavelength of 405 nm. A calibration curve was constructed using known concentrations of *p*-nitrophenol.

#### Detection of Ki-67

Ki-67 is a widely used marker for cell proliferation.^40^ For proliferating cells, the Ki-67 protein is expressed in the active (i.e., late G1, S, G2, and M) stages of the cell cycle. However, Ki-67 protein cannot be detected in the G_0_ stage (i.e., for nonproliferating cells).^60^ Permanox chamber slides were coated with fibronectin at 4.6 μg cm^–2^ to ensure that MSCs adhered to the slide surface.^61^ MSCs were retrieved from the worm gels as described above, added to the wells of the chamber slides, and incubated for 24 h at 37 °C in MSC-CM in a humidified 95% air/5% CO_2_ atmosphere. After 24 h, the culture medium was removed and the attached MSCs were washed with PBS prior to fixing with 4% buffered paraformaldehyde. After fixation, the chamber slides were washed with PBS, sealed with parafilm, and stored at 4 °C for the Ki-67 assay. Chamber slides containing proliferating MSCs were used as a positive control and fixed as described above. The fixed cells were washed with PBS and permeabilized with 0.1% Triton X-100 in PBS for 20 min at 20 °C. The cells were then washed with PBS, and nonspecific binding was blocked by incubating with 10% v/v normal goat serum and 1.0 g dm^–3^ BSA in PBS. The cells were washed with PBS and incubated overnight with the rabbit-anti-human Ki-67 antibody at 4 °C. The chambers were again washed with PBS, and the cells were incubated for 75 min at 20 °C with goat anti-rabbit IgG labeled with Alexa Fluor 488 dye. Then, the chambers were washed with PBS followed by incubation with 0.5 μM DAPI to stain the cell nuclei. The chambers were again washed with PBS prior to their removal, and the slides were mounted using ProLong Diamond Antifade Mountant and examined using a Leica Thunder microscope.

## Results and Discussion

**Figure 1:**
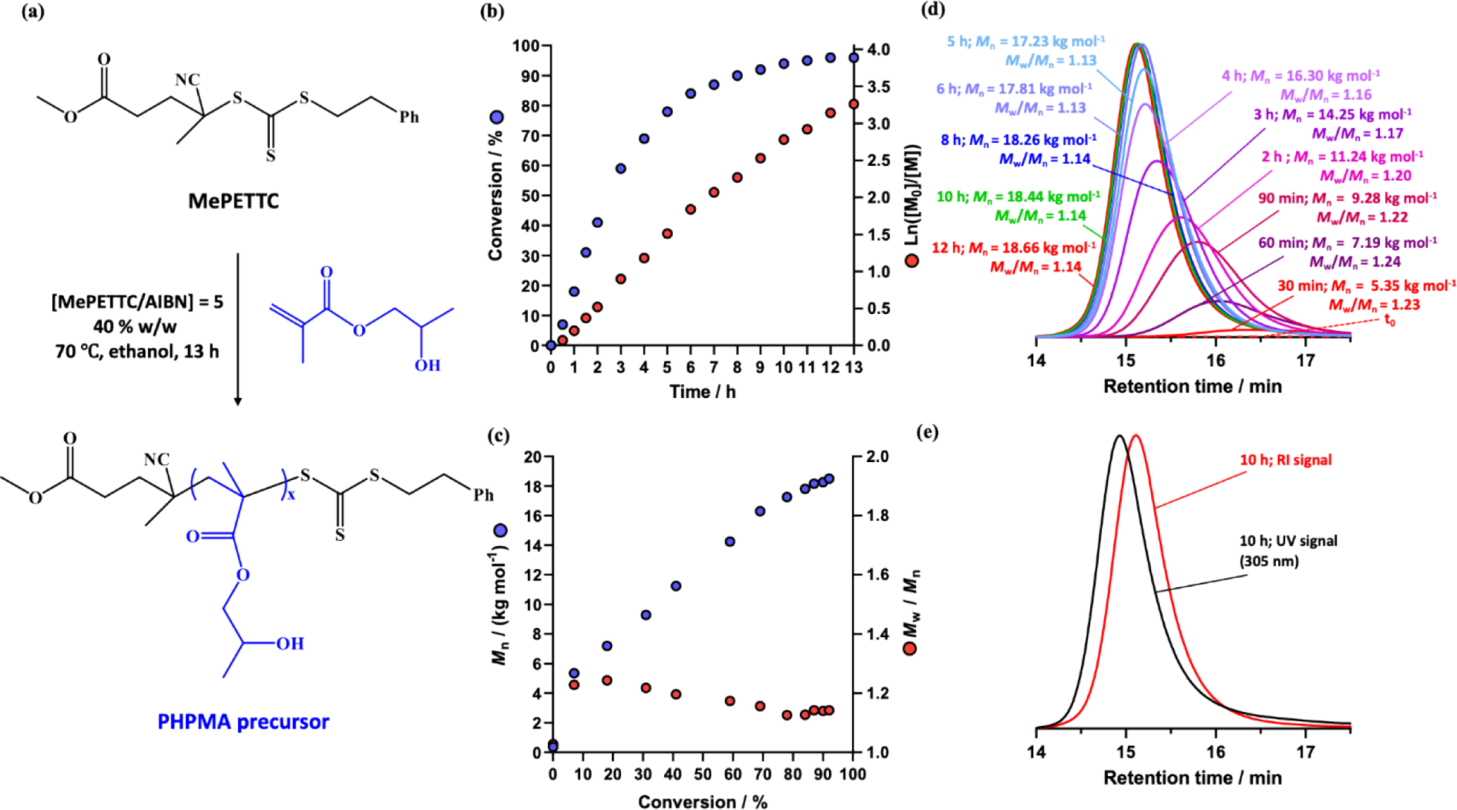
(a) Synthesis route of the PHPMA homopolymer via RAFT solution polymerization of HPMA in ethanol using Me-PETTC RAFT agent at 70 °C. Conditions: 40% w/w solids, AIBN initiator; Me-PETTC/AIBN molar ratio = 5.0; target PHPMA DP = 150. (b) Conversion vs the time curve and corresponding semilogarithmic plot obtained for the polymerization. (c) DMF GPC curves obtained during the course of this HPMA polymerization using a refractive index detector. (d) Corresponding evolution in *M*_n_ and *M*_w_/*M*_n_ with conversion. (e) GPC curves obtained after 10 h using a refractive index detector (red curve) and a UV detector (black curve; λ = 305 nm).

PHPMA was selected as a weakly hydrophobic structure-directing block because it was known that PHPMA-based aqueous worm gels can be readily prepared via direct dissolution in water without recourse to organic solvents.^45^ It is perhaps worth emphasizing that worms most likely correspond to the equilibrium copolymer morphology under such conditions. First, a kinetic study of the RAFT solution polymerization of HPMA in ethanol was conducted using an AIBN initiator at 70 °C (see Figure 1a). In this experiment, the mass of MePETTC was adjusted to target a mean DP of 150. This technique was used to determine the HPMA DP by comparing the MePETTC RAFT CTA aromatic signals at 7.5–7.1 ppm to the methacrylic backbone signals at 1.7–2.46 ppm after purification (see Figure 1b). Representative ^1^H NMR spectra for MePETTC and the purified PHPMA precursor are shown in Figure S1. DMF GPC (refractive index detector) was used to monitor the evolution in *M*_n_ and *M*_w_/*M*_n_ during this kinetic study (see Figure 1c), while UV GPC was employed to confirm retention of the RAFT end-groups after the HPMA polymerization (see Figure 1e). The evolution in *M*_n_ and *M*_w_/*M*_n_ during the RAFT solution polymerization of HPMA in ethanol at 70 °C is shown in Figure 1d. As expected for a well-controlled RAFT polymerization,^2,62^ relatively low copolymer dispersities (*M*_w_/*M*_n_ < 1.20) were obtained and a monotonic increase in *M*_n_ was observed with HPMA conversion (with an approximately linear increase up to around 80% conversion). To maximize retention of the trithiocarbonate end-groups, all subsequent polymerizations were terminated at approximately 80% HPMA conversion (which required reaction times of around 6 h at 70 °C).

**Figure 2:**
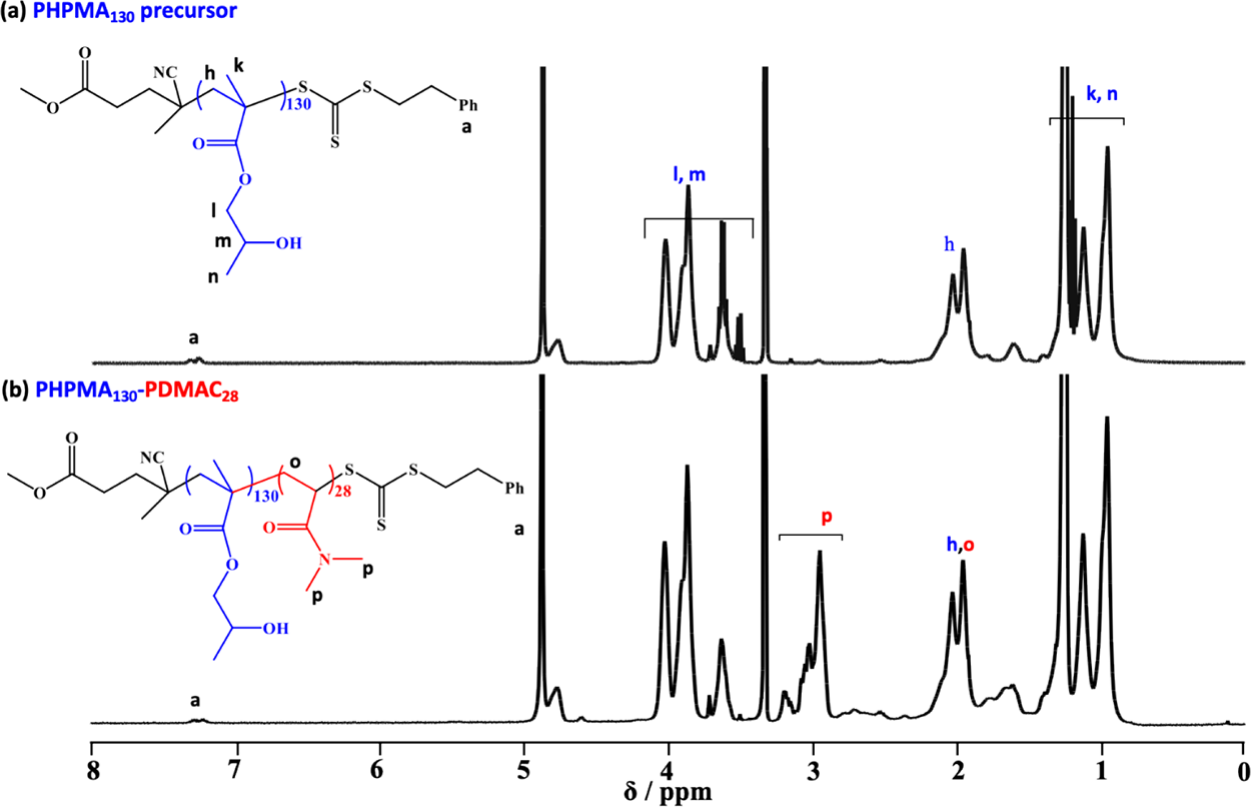
^1^H NMR spectra recorded in CD_3_OD for (a) a PHPMA_130_ precursor and (b) the corresponding PHPMA_130_-PDMAC_28_ diblock copolymer.

**Scheme 1:**
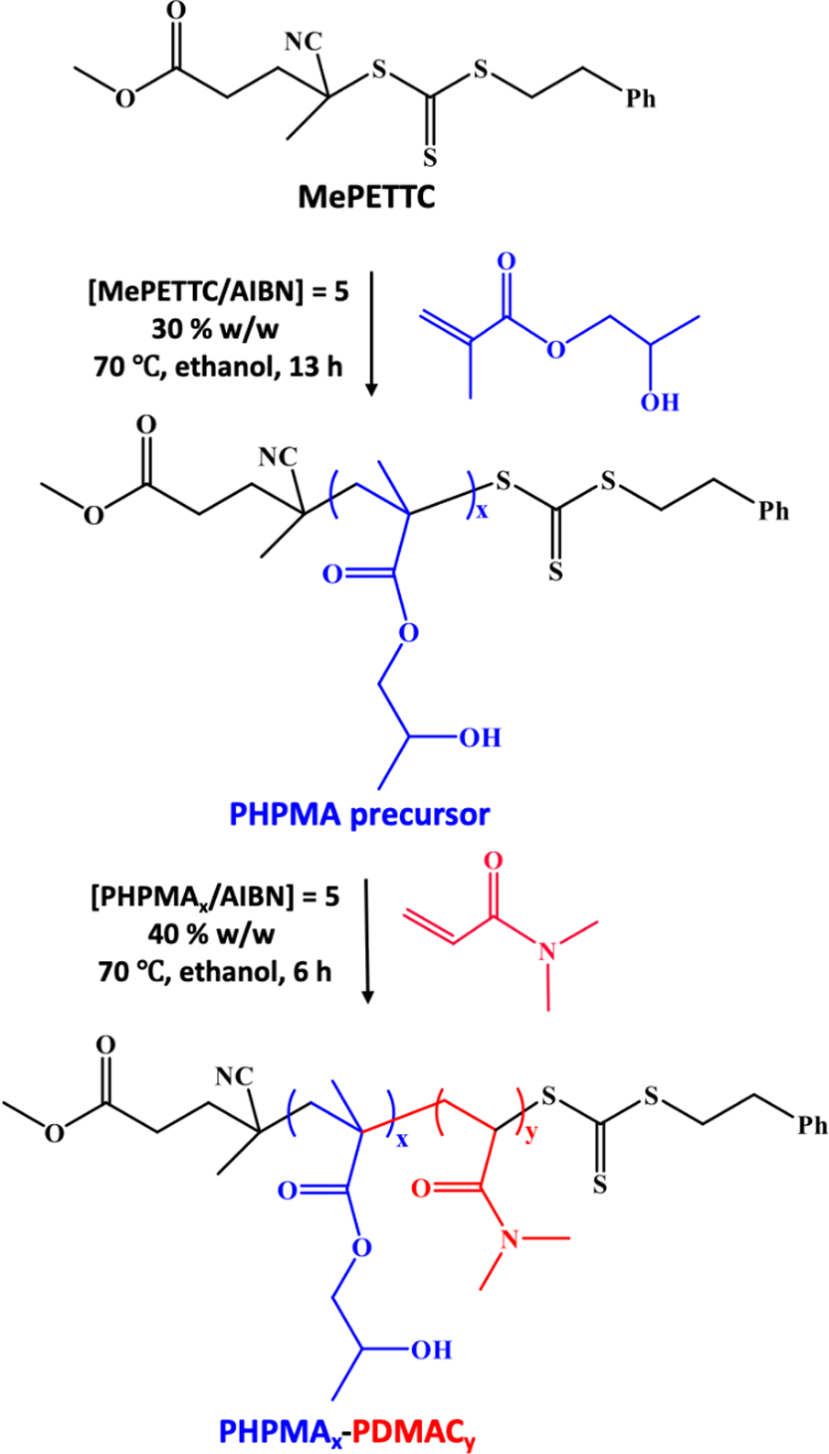
Synthesis of PHPMA_*x*_-PDMAC_*y*_ Diblock Copolymers by RAFT Solution Polymerization of *N*,*N*-Dimethylacrylamide (DMAC) Using a PHPMA_*x*_ Precursor and AIBN Initiator in Ethanol at 70 °C

Accordingly, near-monodisperse PHPMA_101_, PHPMA_130_, and PHPMA_148_ precursors were prepared via RAFT solution polymerization in ethanol at 70 °C. These three precursors were then used to prepare several series of PHPMA_*x*_-PDMAC_*y*_ diblock copolymers (see Scheme 1). Molecular weight data were obtained by ^1^H NMR spectroscopy studies and DMF GPC analysis. The mean DP for each PHPMA_*x*_-PDMAC_*y*_ diblock copolymer was calculated from the DMAC conversion using the PHPMA block as an “end-group” by comparing the methylene methacrylic backbone protons (2H, 1.18–2.26 ppm) to the dimethyl protons assigned to PDMAC (6H, 2.29–3.36 ppm), see Figure 2.

**Figure 3:**
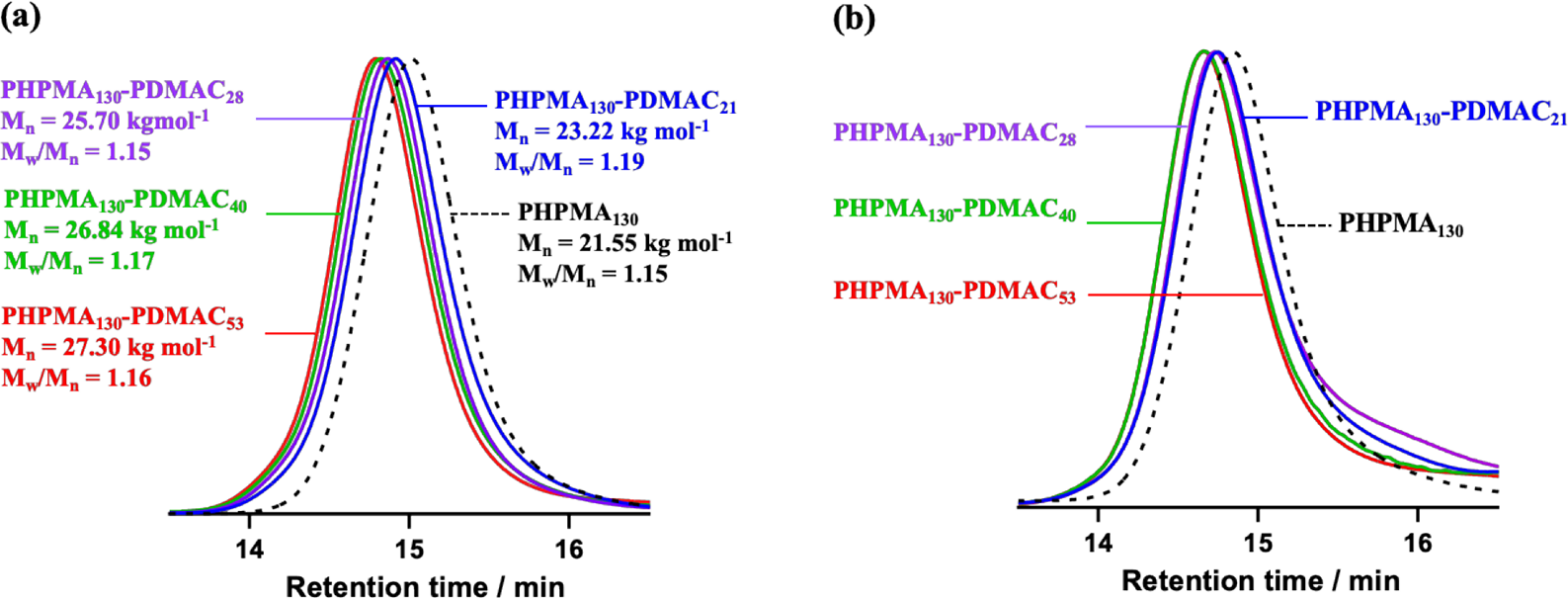
(a) DMF GPC curves obtained for a series of five PHPMA_130_-PDMAC_*y*_ diblock copolymers and the corresponding PHPMA_130_ precursor (dashed trace) using a refractive index detector. (b) UV GPC curves (λ = 305 nm) obtained for three PHPMA_130_-PDMAC_*y*_ diblock copolymers and the corresponding PHPMA_130_ precursor (dashed trace).

DMF GPC curves recorded for a PHPMA_130_ precursor and corresponding PHPMA_130_-PDMAC_*y*_ diblock copolymers are shown in Figure 3. Relatively low dispersities (*M*_w_/*M*_n_ < 1.20) and a proportionate increase in *M*_n_ were observed for all copolymers. Moreover, UV GPC studies indicated that trithiocarbonate end-groups were retained after the DMAC polymerization. Similar results were obtained when using a PHPMA_141_ precursor to prepare a PHPMA_141_-PDMAC_36_ diblock copolymer, see Table 1.

**Scheme 2:**
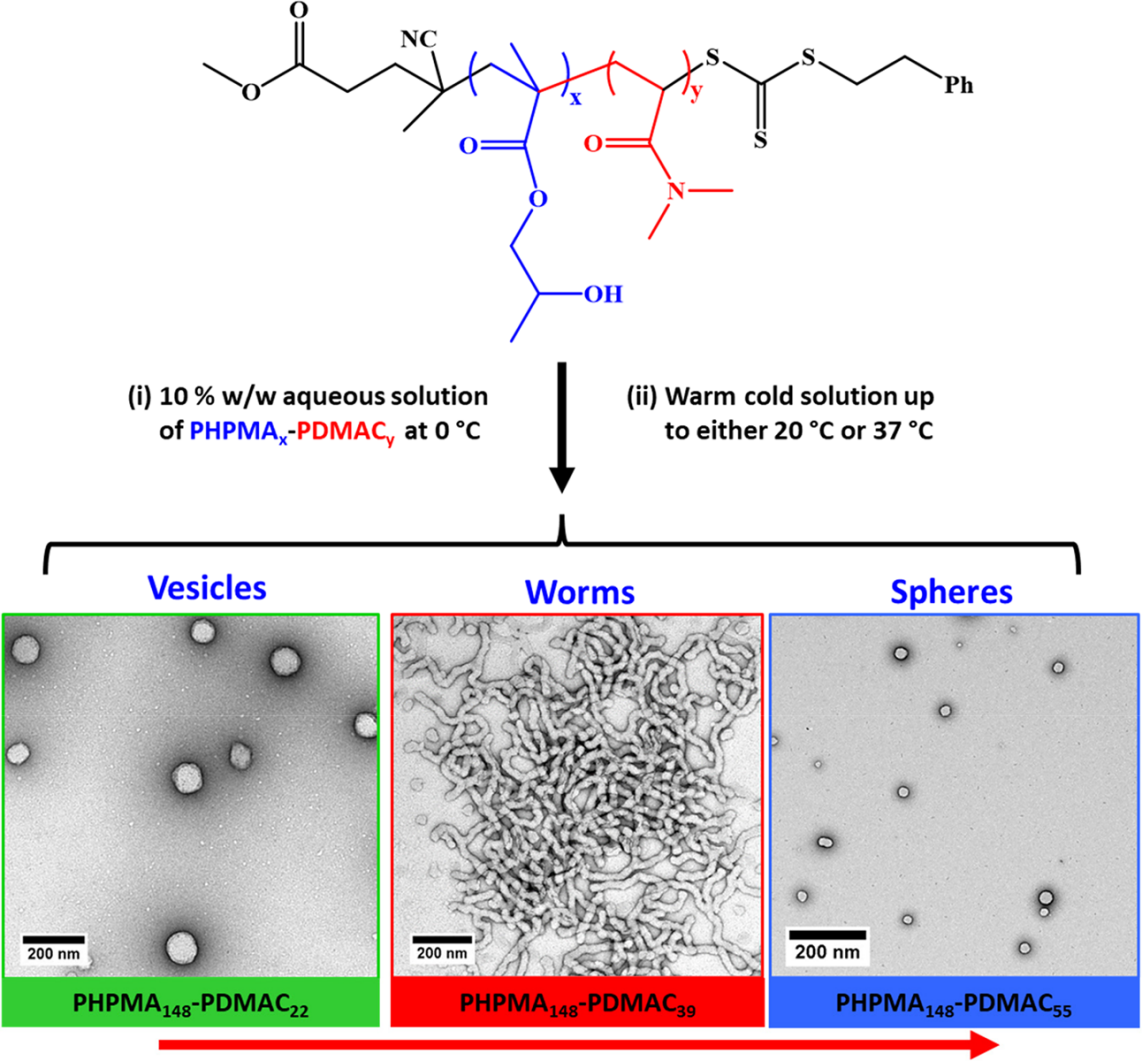
Direct Dissolution of Amphiphilic PHPMA-PDMAC Diblock Copolymers in Ice-Cold Water Affords Spheres, Worms, or Vesicles at 10% w/w Solids via *in situ* Self-Assembly on Warming to 37 °C The precise diblock copolymer composition dictates the final morphology.

Next, such amphiphilic PHPMA-PDMAC diblock copolymers were dissolved directly in ice-cold water to induce self-assembly and hence afford sterically stabilized nano-objects. Depending on the precise diblock copolymer composition, TEM studies indicated that this protocol produced spheres, worms, or vesicles at 10% w/w solids (Scheme 2).

**Figure 4:**
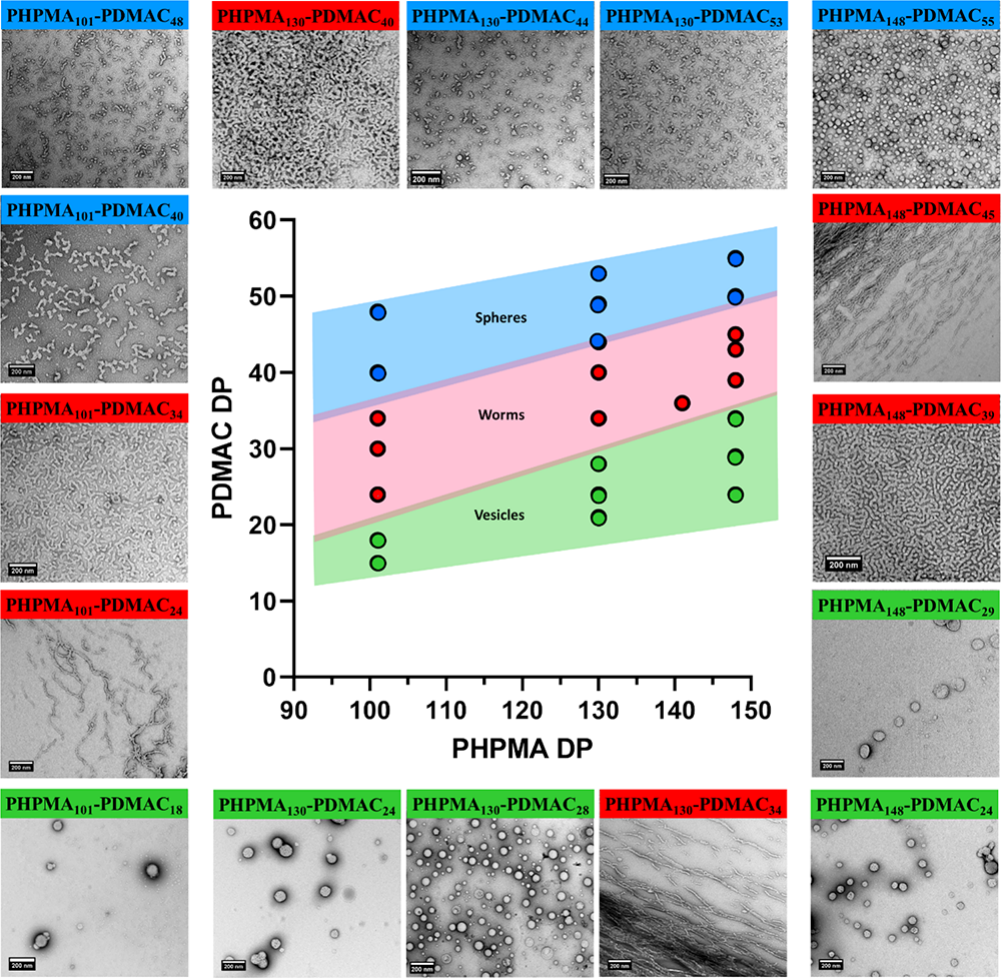
Pseudo-phase diagram for three series of PHPMA_101_-PDMAC_*y*_, PHPMA_130_-PDMAC_*y*_, and PHPMA_148_-PDMA_*z*_ diblock copolymer nano-objects prepared via direct dissolution at 10% w/w solids in aqueous media at 37 °C. All morphological assignments were made on the basis of TEM studies (see selected TEM images).

Our prior studies provide useful guidance for selecting the likely optimum PHPMA DP.^11,28^ However, it is nevertheless challenging to target a pure PHPMA-PDMAC worm gel phase. This is because the worm morphology requires PHPMA-rich diblock compositions (typically around 75–80 mol %). Thus, for a given PHPMA DP, only a very limited range of PDMAC DPs will produce a pure worm phase. Accordingly, the construction of a detailed pseudo-phase diagram using three series of 10% w/w PHPMA_*x*_-PDMAC_*y*_ diblock copolymers is essential to identify the optimum PDMAC DP. TEM was employed to assign the copolymer morphology (see Figure 4). For a fixed PHPMA DP, targeting a higher PDMAC DP leads to a change in copolymer morphology from vesicles to worms to spheres as the volume fraction of the hydrophilic steric stabilizer block is increased. Similarly, for a fixed PDMAC DP, increasing the PHPMA DP leads to a change in copolymer morphology either from spheres to worms or from worms to vesicles. Both sets of observations can be rationalized in terms of the geometric packing parameter initially introduced by Israelachvili and co-workers to account for surfactant self-assembly^63,64^ and subsequently applied to diblock copolymer self-assembly.^65^

Clearly, the worm phase space is relatively narrow, particularly when targeting a PHPMA DP above 130. More specifically, for a PHPMA_148_-PDMAC_*x*_ diblock copolymer, *x* can only vary by around 5–6 repeat units before mixed phases are formed instead of the desired pure worm phase.^11^ In contrast, targeting a PHPMA DP of 101 provides somewhat broader phase space, with pure worms being obtained for PDMAC DPs ranging from 24 to 34.

**Figure 5:**
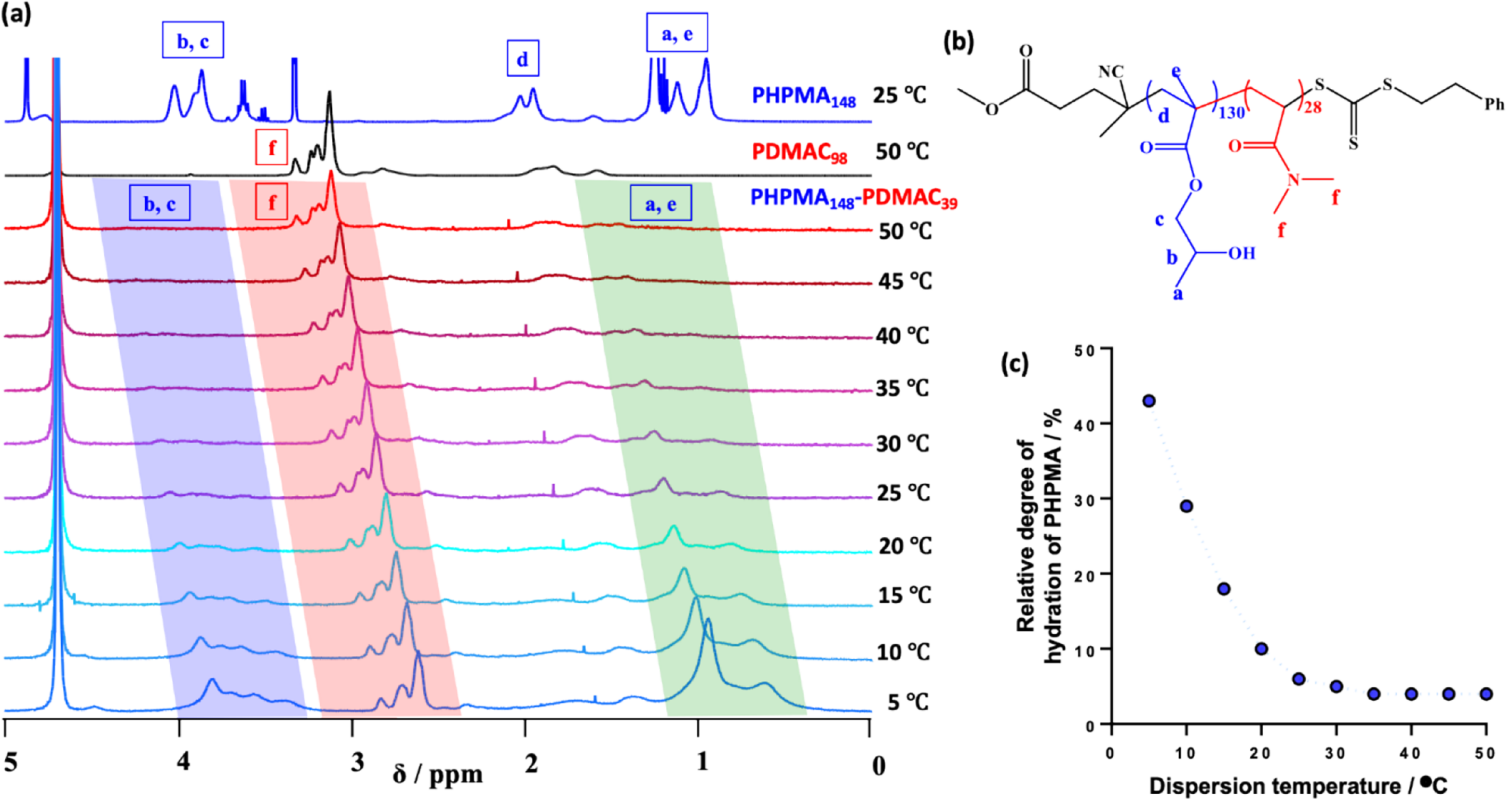
(a) Variable temperature ^1^H NMR studies of a 10% w/w dispersion of PHPMA_148_-PDMAC_39_ diblock copolymer nano-objects in D_2_O, along with reference spectra for a 10% w/w solution of the PHPMA_148_ homopolymer in CD_3_OD at 20 °C, and a 10% w/w solution of the PDMAC_98_ homopolymer in D_2_O at 50 °C. (b) Chemical structure of a PHPMA_148_-PDMAC_39_ diblock copolymer indicating the assigned proton signals shown in panel a. (c) Relative apparent degree of hydration calculated for the hydrophobic PHPMA_39_ block as a function of temperature from the spectra shown in panel a.

The thermoresponsive behavior exhibited by the new amphiphilic PHPMA-PDMAC diblock copolymers reported herein is driven by the variable degree of hydration of the weakly hydrophobic structure-directing PHPMA chains.^37,66−68^ Accordingly, variable temperature ^1^H NMR spectra were recorded from 5 up to 50 °C (see Figure 5). At 5 °C, the PHPMA chains had an apparent degree of hydration of more than 40%, but this was reduced to 5% at 30 °C. We have reported similar—but much more qualitative—observations for other PHPMA-based amphiphilic diblock copolymers.^30−37^ In these prior studies, the main technical problem was overlap between the PHPMA proton signals and those of the steric stabilizer chains. This issue does not exist for the PHPMA-PDMAC diblock copolymer system. Indeed, the data set shown in Figure 5 is by far the most convincing evidence for the thermoresponsive nature of the weakly hydrophobic PHPMA chains yet reported.

The critical gelation concentration (CGC) of PHPMA_148_-PDMAC_39_ worms was determined by conducting oscillatory rheology studies at 20 and 37 °C on a series of PHPMA_148_-PDMAC_39_ worm dispersions of varying concentrations at a copolymer concentration of 10% w/w, the storage modulus, *G*′ and was determined to be approximately 60 Pa at 20 °C and 80 Pa 37 °C, respectively (Figure S2a,b). On lowering the copolymer concentration to 6% w/w, *G*″ becomes equal to *G*′ at both temperatures, so this concentration corresponds to the CGC. Only free-flowing fluids (*G*′ < 1 Pa) were obtained below 6% w/w. Moreover, the thermoresponsive behavior of a 10% w/w aqueous dispersion of PHPMA_148_-PDMAC_39_ worms in the presence or absence of PBS was studied by oscillatory rheology during a 5 to 37 to 5 °C thermal cycle (Figure S2c,d). The initial *G*′ at 5 °C was approximately 0.1 Pa in pure water and 3 Pa in the presence of PBS. On heating, *G*″ became equal to *G*′ at around 18 °C, which corresponds to the critical gelation temperature (CGT).

At 37 °C, a free-standing worm gel was obtained with a *G*′ of approximately 60 Pa. Good thermoreversibility was observed during the cooling cycle in the absence of PBS. However, a much higher *G*′ of ∼5000 Pa was obtained at 37 °C in the presence of PBS. Moreover, in this case *G*′ and *G*″ did not regain their initial values during the cooling cycle. In view of these observations, the concentration of PHPMA_148_-PDMAC_39_ worms in PBS was reduced to 5% w/w for further studies.

**Figure 6:**
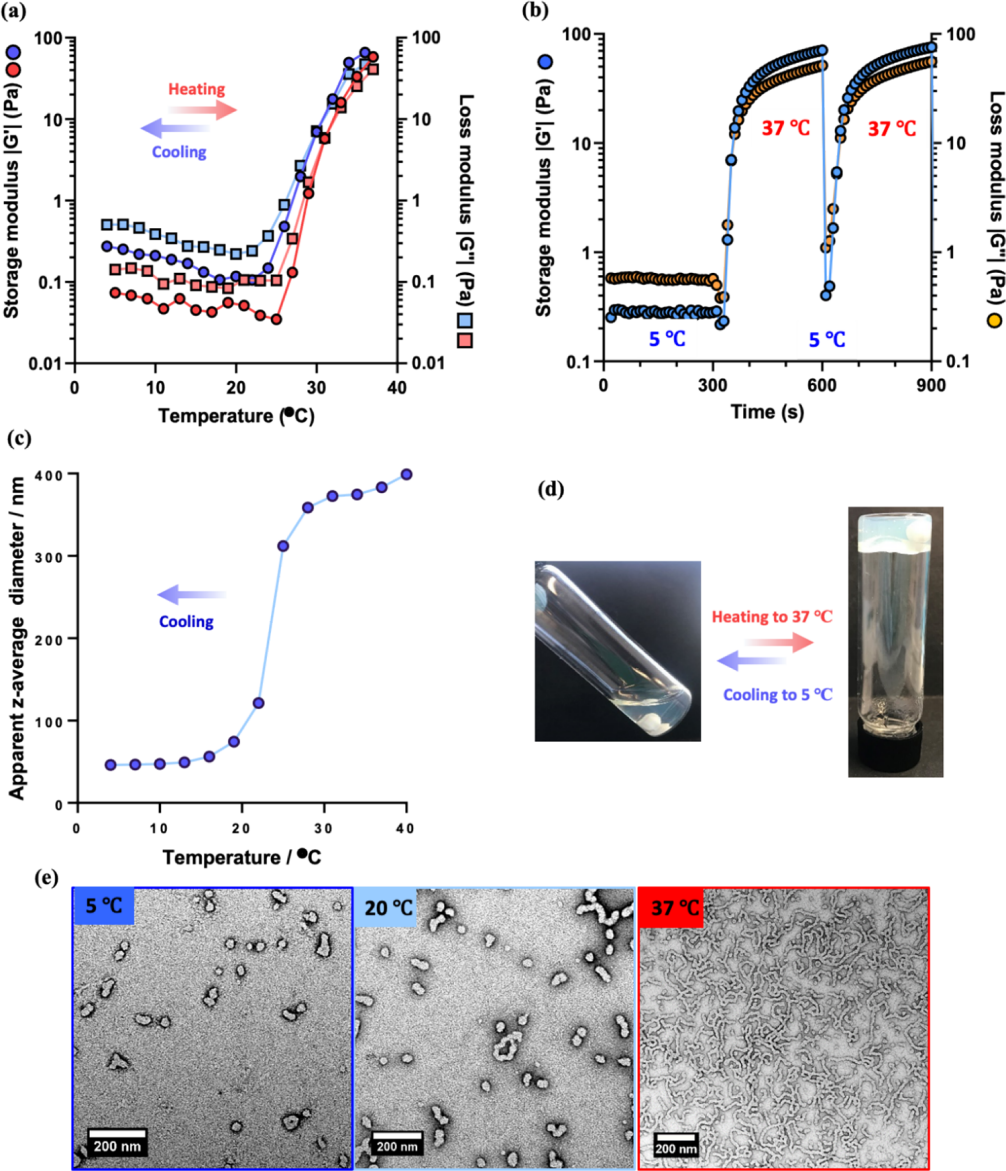
Variable temperature oscillatory rheology data obtained for a 10% w/w aqueous dispersion of PHPMA_141_-PDMAC_36_ worms in deionized water: (a) during a 5 to 37 to 5 °C thermal cycle (heating *G*′ and *G*″ data are denoted by red circles and squares, respectively, while cooling *G*′ and *G*″ data are denoted by blue circles and squares, respectively). (b) Temperature jump experiments from 5 to 37 to 5 °C (*G*′ data are denoted by blue circles, while *G*″ data are denoted by orange circles). (c) DLS data obtained for a 0.1% w/w aqueous dispersion of PHPMA_141_-PDMAC_36_ worms on cooling from 40 to 4 °C. (d) Digital photographs obtained for a 10% aqueous dispersion of PHPMA_141_-PDMAC_36_ worms at 5 and 37 °C. (e) TEM images recorded after drying a 0.03% w/w aqueous dispersion of PHPMA_141_-PDMAC_36_ worms at 5, 20, or 37 °C. Oscillatory rheology studies were conducted using a strain of 1.0% and an angular frequency of 1.0 rad s^–1^.

A second copolymer was also examined in an attempt to obtain more thermoreversible worms in the presence and absence of PBS. Accordingly, the thermoresponsive behavior of a 10% w/w aqueous dispersion of PHPMA_141_-PDMAC_36_ worms was investigated by oscillatory rheology during a 5 to 37 to 5 °C thermal cycle (Figure 6a). The *G*′ of the initial 10% w/w aqueous dispersion of spheres at 5 °C was determined to be approximately 0.1 Pa. On heating, *G*″ became equal to *G*′ at around 30 °C, which corresponds to the critical gelation temperature (CGT). After maintaining an initial constant temperature of 5 °C for 5 min, temperature jump experiments were conducted via rapid heating from 5 to 37 °C and rapid cooling from 37 to 5 °C (Figure 6b). In this case, the initial *G*′ for a 10% w/w dispersion of PHPMA_141_-PDMAC_36_ spheres was determined to be approximately 0.3 Pa at 5 °C. In contrast, the worms formed on rapidly heating to 37 °C exhibited a *G*′ of approximately 60 Pa. Very similar *G*′ values were observed during the second temperature jump experiment, indicating good reproducibility. Furthermore, the thermoresponsive behavior of a 0.1% w/w aqueous dispersion of PHPMA_141_-PDMAC_36_ worms was investigated by DLS while cooling from 40 to 4 °C (Figure 6c). The apparent *z*-average diameter of the initial worms was approximately 400 nm at 40 °C but only around 50 nm at 4 °C. This is reasonably consistent with the expected worm-to-sphere transition.^51^ Digital photographs recorded for a 10% w/w aqueous dispersion of PHPMA_141_-PDMAC_36_ worms confirmed the formation of a free-flowing fluid at 4 °C and a soft free-standing worm gel at 37 °C (Figure 6d). Moreover, TEM studies of a 0.03% w/w aqueous dispersion of PHPMA_141_-PDMAC_36_ nano-objects dried at 37, 20, or 5 °C, respectively, indicated the presence of highly anisotropic worms at 37 °C, a mixed phase comprising short worms and spheres at 20 °C, and mainly spheres (plus a few dimers and trimers) at 5 °C (Figure 6e). Similar observations were reported by Blanazs et al. for PGMA-PHPMA worms.^38^

**Figure 7:**
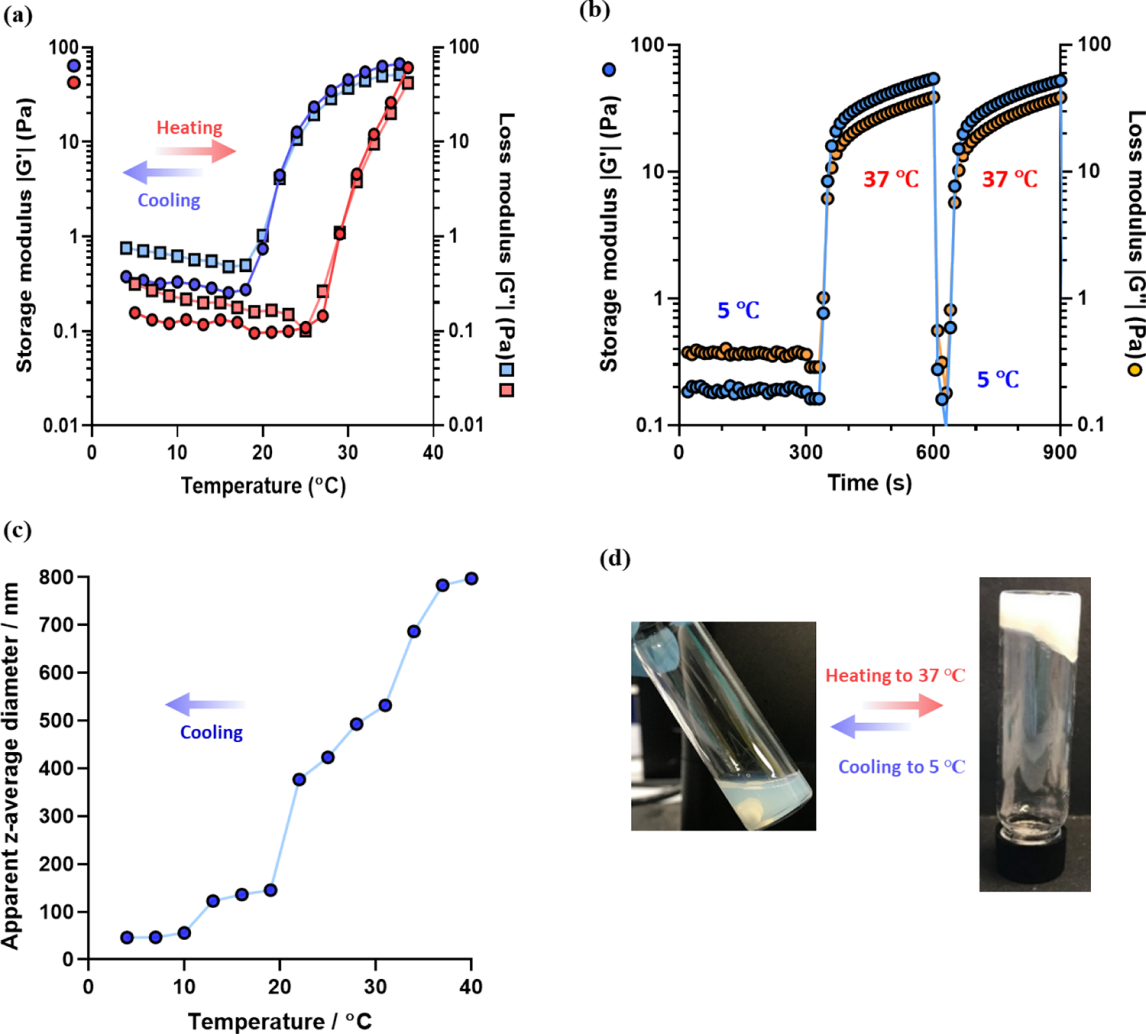
Variable temperature oscillatory rheology data obtained for a 5% w/w aqueous dispersion of PHPMA_141_-PDMAC_36_ worms in the presence of PBS: (a) during a 5 to 37 to 5 °C thermal cycle (heating *G*′ and *G*″ data are denoted by red solid circles and squares, respectively; cooling *G*′ and *G*″ data are denoted by blue solid circles and squares, respectively). Temperature jump experiments from 5 to 37 to 5 °C (*G*′ data are denoted by blue circles, while *G*″ data are denoted by orange circles). (c) DLS data obtained for a 0.1% w/w aqueous dispersion of PHPMA_141_-PDMAC_36_ worms on cooling from 40 to 4 °C. (d) Digital images recorded for a 10% w/w aqueous dispersion of PHPMA_141_-PDMAC_36_ nano-objects at 5 °C (free-flowing fluid) and 40 °C (soft free-standing gel).

Finally, a 5% w/w aqueous dispersion of PHPMA_141_-PDMAC_36_ worms in the presence of PBS was studied by oscillatory rheology during a 5 to 37 to 5 °C thermal cycle (Figure 7a).

The *G*′ of the initial spheres formed at 5 °C was determined to be approximately 0.1 Pa. During the heating cycle, *G*″ becomes equal to *G*′ at around 30 °C, which corresponds to the CGT. *G*′ is related to the gel strength, and previous studies suggested that a *G*′ of less than 100 Pa is optimal for effective stem cell storage.^39,40^ Gratifyingly, *G*′ was determined to be around 70 Pa at 37 °C.

Again, temperature jump experiments were conducted from 5 to 37 to 5 °C after maintaining an initial temperature of 5 °C for 5 min (Figure 7b). In this case, the 5% w/w aqueous dispersion of PHPMA_141_-PDMAC_36_ nano-objects exhibited an initial *G*′ of approximately 0.2 Pa in the presence of PBS at 5 °C. After rapidly heating to 37 °C, *G*′ increased up to approximately 70 Pa within 300 s. Similar *G*′ values were obtained in a second temperature jump experiment. Furthermore, the thermoresponsive behavior of an 0.1% w/w aqueous dispersion of PHPMA_141_-PDMAC_36_ nano-objects was investigated by DLS while cooling from 40 to 4 °C (Figure 7c). In this case, the apparent *z*-average diameter of the initial worms was approximately 800 nm at 40 °C and around 50 nm at 4 °C. Digital photographs recorded for a 5% w/w aqueous dispersion of PHPMA_141_-PDMAC_36_ worms confirm formation of a free-flowing fluid at 4 °C and a soft free-standing worm gel at 37 °C (Figure 7d).

**Figure 8:**
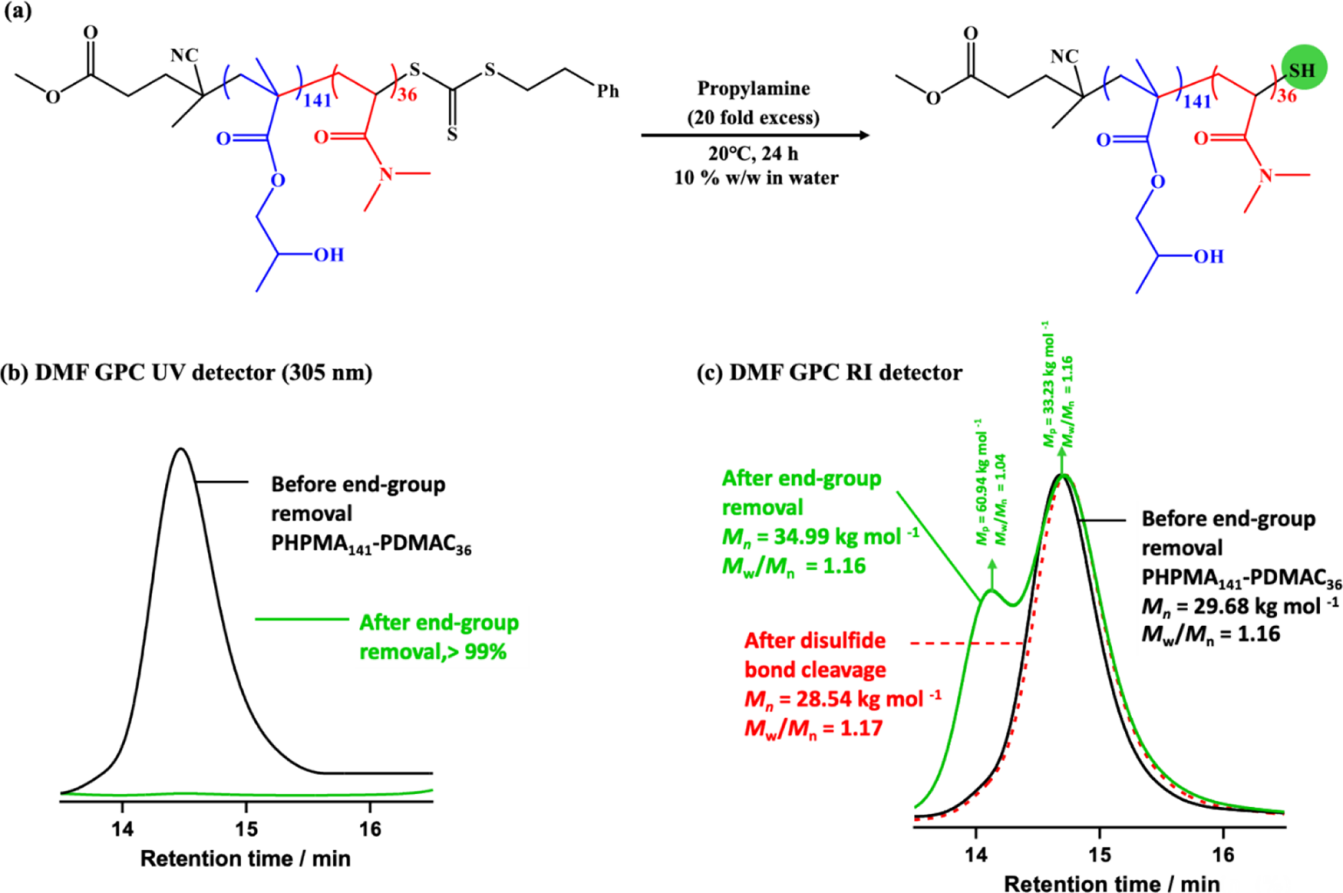
(a) Reaction scheme for the selective removal of the trithiocarbonate-based RAFT end-group from an aqueous dispersion of PHPMA_141_-PDMAC_36_ worms at 20 °C using a 20-fold excess of propylamine relative to the number of moles of end-groups. (b) DMF GPC curves recorded for PHPMA_141_-PDMAC_36_ using a UV detector (λ = 305 nm) data to assess the extent of end-group removal. The lower green curve suggests that more than 99% of the trithiocarbonate end-groups are removed after treatment with excess propylamine. (c) DMF GPC curves (refractive index detector) recorded for the PHPMA_141_-PDMAC_36_ diblock copolymer before and after end-group removal, before end group removing (solid black curve), after end-group removal (solid green curve), and after disulfide bond cleavage (dash red curve).

In principle, removal of the RAFT chain-ends from the PHPMA_141_-PDMAC_36_ worms should be straightforward because (i) they are located at the end of the soluble steric stabilizer block (rather than buried within the worm cores) and (ii) an acrylamide-based terminal unit is less sterically congested—and hence more reactive—than the analogous methacrylic terminal unit. Accordingly, a 10% w/w aqueous dispersion of PHPMA_141_-PDMAC_36_ worms was treated with a 20-fold excess of propylamine at 20 °C to remove the trithiocarbonate end-groups (Figure 8a). DMF GPC studies (refractive index detector) indicated some broadening of the molecular weight distribution, with a modest increase in *M*_n_ and *M*_w_/*M*_n_. Importantly, DMF GPC analysis using a UV detector (λ = 305 nm) indicated that more than 99% of the trithiocarbonate end-group were removed (Figure 8b).

In particular, a new high-molecular-weight shoulder is discernible, which suggests the formation of a fraction of PHPMA_141_-PDMAC_36_-S-S-PDMAC_36_-PHPMA_141_ triblock copolymer chains via thiol-thiol coupling (Figure 8c). This interpretation is supported by the observation that the *M*_p_ estimated for this shoulder is approximately twice that of the GPC peak for the diblock copolymer chains (i.e., 60 kg mol^–1^ vs 33 kg mol^–1^). To verify this hypothesis, the copolymer chains were treated with a 100-fold excess of DTT, which is known to cleave the disulfide bond (see Scheme S3).^68−70^ Furthermore, the high-molecular-weight shoulder disappears after such DTT treatment. Visual inspection of freeze-dried PHPMA_141_-PDMAC_36_ powder before and after end-group removal confirmed the expected color change from pale yellow to white (Figure S4).

**Figure 9:**
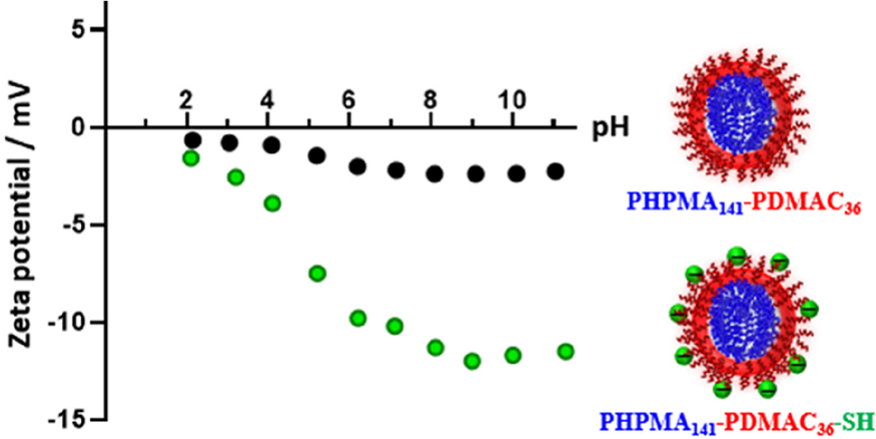
Zeta potential vs curves obtained for PHPMA_141_-PDMAC_36_ (black data set) and PHPMA_141_-PDMAC_36_–SH nano-objects (green data set). In the latter case, ionization of the terminal thiol groups located at the end of the stabilizer chains leads to appreciable anionic character in alkaline media.

In addition, ^1^H NMR spectroscopy was also employed to assess the extent of end-group removal. More specifically, the aromatic proton signals observed at 7.1–7.4 ppm for the PHPMA_141_-PDMAC_36_ precursor were no longer visible for the propylamine-treated diblock copolymer (Figure S5). Furthermore, aqueous electrophoresis studies were performed to examine the effect of varying the solution pH on the electrophoretic footprint of the thiol-functionalized PHPMA_141_-PDMAC_36_ nano-objects at 20 °C, see Figure 9.

The zeta potential obtained for the PHPMA_141_-PDMAC_36_ nano-objects was approximately −2 mV at pH 11, as expected for the nonionic PDMAC steric stabilizer chains. In contrast, the PHPMA_141_-PDMAC_36_–SH nano-objects exhibited a zeta potential of approximately −12 mV under the same conditions, indicating ionization of the terminal thiol groups. Similar observations have been made for other sterically stabilized nanoparticles bearing single ionizable groups located at the end of the steric stabilizer chains.^19^ In principle, such terminal thiol groups should enable facile conjugation of desirable functional groups such as fluorescent dyes or oligopeptides to these nanoparticles.

**Figure 10:**
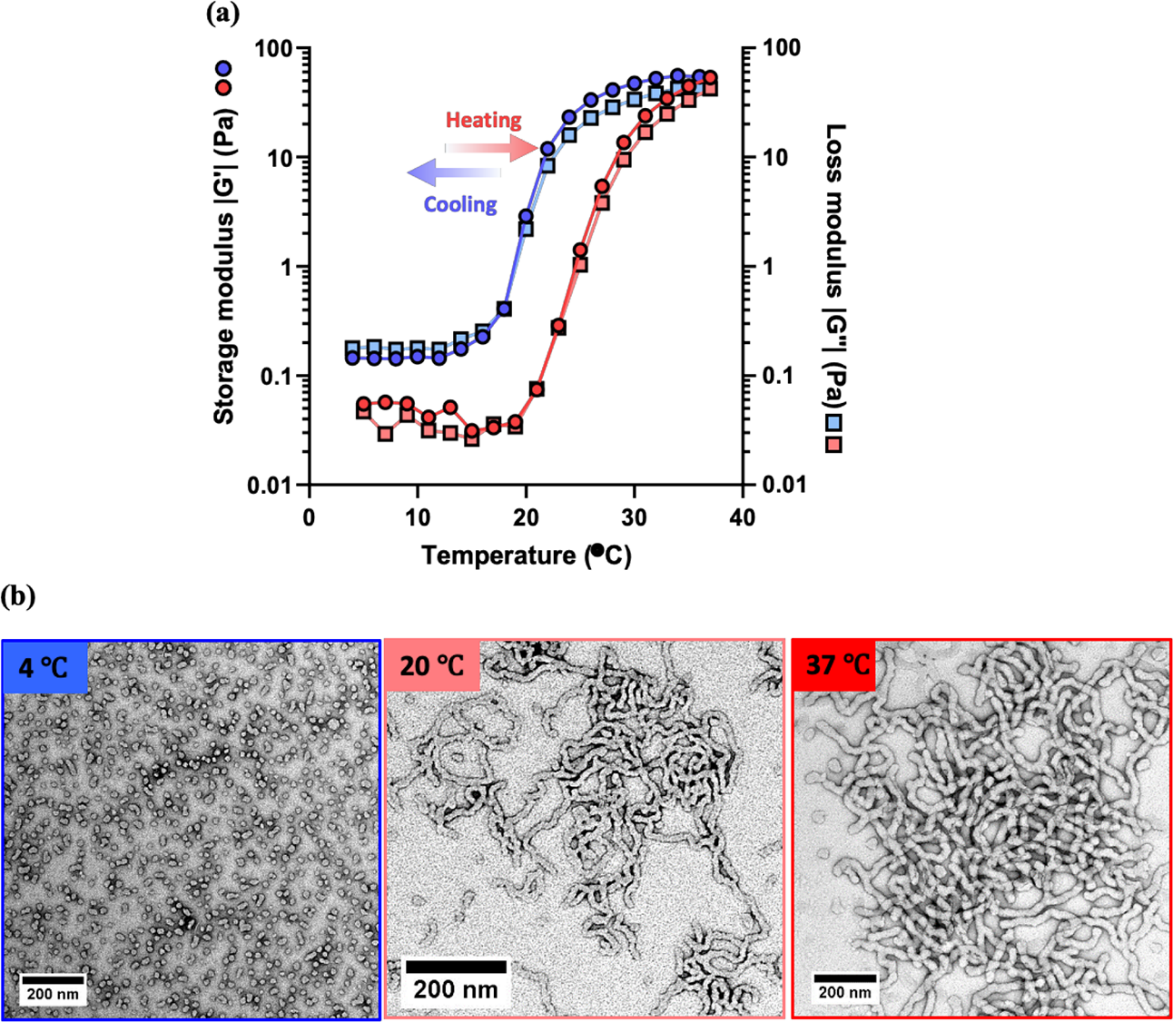
Variable temperature oscillatory rheology data obtained for a 10% w/w aqueous dispersion of PHPMA_141_-PDMAC_36_ worms in the presence of PBS: (a) during a 5 to 37 to 5 °C thermal cycle (heating *G*′ and *G*″ data denoted by red solid circles and squares, respectively; cooling *G*′ and *G*″ data denoted by blue solid circles and squares, respectively). These oscillatory rheology studies were conducted at 1.0% and an angular frequency of 1.0 rad s^–1^. (b) TEM images recorded after drying a 0.03% w/w aqueous dispersion of PHPMA_141_-PDMAC_36_ nano-objects at either 5 °C (spheres) or 37 °C (worms).

After RAFT end-group removal, the thermoresponsive behavior of a 5% w/w aqueous dispersion of PHPMA_141_-PDMAC_36_-SH worms in the presence of PBS was investigated by oscillatory rheology during a 5 to 37 to 5 °C thermal cycle (Figure 10a).

The *G*′ value for the PHPMA_141_-PDMAC_36_-SH worm gel was determined to be approximately 70 Pa at 37 °C. TEM studies indicated the presence of worms at 37 °C, a mixture of worms and spheres at 20 °C, and spheres at 5 °C in aqueous (Figure 10b). Under the latter conditions, a *G*′ of 0.05 Pa was obtained for the free-flowing fluid at 5 °C.

Cytocompatibility was assessed by encapsulating MSCs within 4% w/w PHPMA_141_-PDMAC_36_ or 6% w/w PGMA_55_-PHPMA_135_ worm gels, respectively. Each 150 μL aliquot of worm gel contained 150,000 MSCs and was cultured for 1–3 weeks on 2% agarose-coated 24-well plates to prevent cell attachment to the surface of the culture plate.

**Figure 11:**
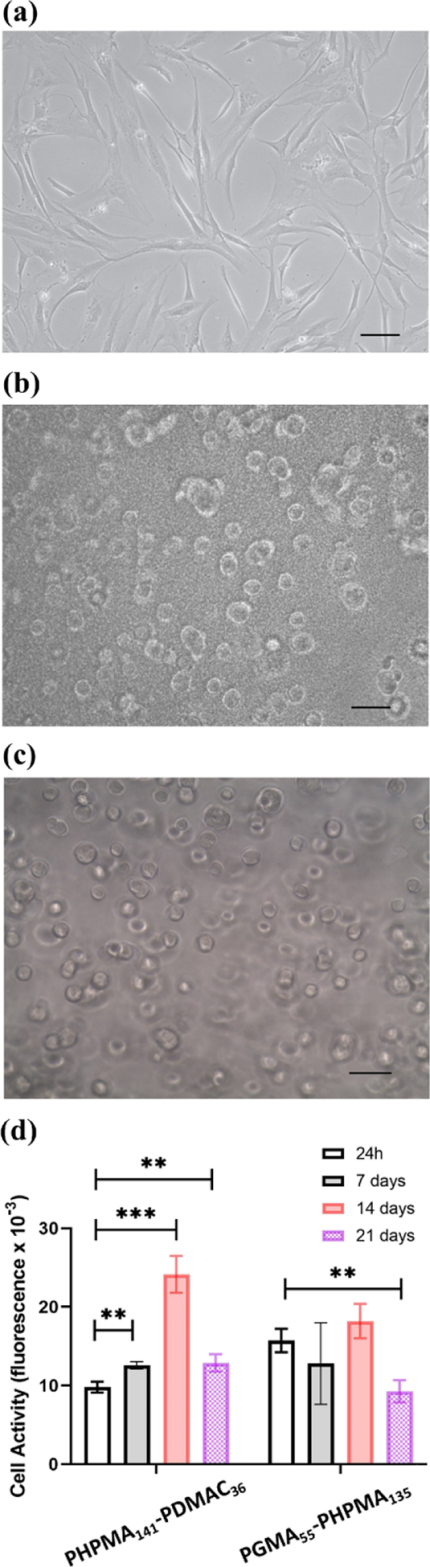
Phase contrast light microscopy images recorded for hBM-MSCs stem cells and cell viability data obtained after MSC encapsulation within worm gels for 1–3 weeks at 37 °C. Optical microscopy images recorded for: (a) a monolayer culture of hBM-MSC cells; (b) MSCs encapsulated within a 4% w/w PHPMA_141_-PDMAC_36_ worm gel; (c) MSCs encapsulated within a 6% w/w PGMA_55_-PHPMA_135_ worm gel. (d) Cell viability data (PrestoBlue assay) obtained for MSCs within worm gels indicates cell metabolic activity normalized relative to a control sample of the culture medium incubated without any cells. (Scale bars = 50 μm; ns: nonsignificant, * *P* ≤ 0.05, ** *P* ≤ 0.01, and *** *P* ≤ 0.001).

When cultured as monolayers, MSCs exhibit a characteristic fibroblast-type morphology (Figure 11a). However, when encapsulated within 4% w/w PHPMA_141_-PDMAC_36_ or 6% w/w PGMA_55_-PHPMA_135_ worm gels, a rounded cell phenotype was observed within the worm gels (Figure 11b,c). After encapsulation for 24 h, the metabolic activity of the encapsulated MSCs was reduced to 10.0 ± 5.0% in the PHPMA_141_-PDMAC_36_ gel and 14.1 ± 6.7% in the PGMA_55_-PHPMA_135_ gel compared to that of MSCs in the monolayer culture (100 ± 6.0%). Viable MSC cells were observed throughout the three-week encapsulation period for both worm gels. The viability of the encapsulated cells was assessed by measuring their metabolic activity using PrestoBlue during their encapsulation for up to three weeks at 37 °C (Figure 11d). MSCs encapsulated within the PHPMA_141_-PDMAC_36_ worm gel exhibited a small but significant increase (*P* ≤ 0.001) in cellular metabolic activity on day 7 compared to day 1. There was another significant increase in cell metabolic activity by day 14. However, this was reduced to that of day 1 within 21 days. For comparison, MSCs encapsulated within the PGMA_55_-PHPMA_135_ worm gel did not exhibit any significant changes in cellular metabolic activity over the first 14 days This observation is consistent with our earlier report of stasis induction for such hydroxyl-rich worm gels.^39,40^ However, a significant reduction (*P* ≤ 0.001) in metabolic activity was subsequently observed between days 14 and 21 for the PGMA_55_-PHPMA_135_ worm gels. A reduction in cellular metabolic activity between day 14 and day 21 was also observed for MSCs encapsulated in the 4% w/w PHPMA_141_-PDMAC_36_ gel. This suggests that the gel environment is no longer optimal for the encapsulated MSCs.

**Figure 12:**
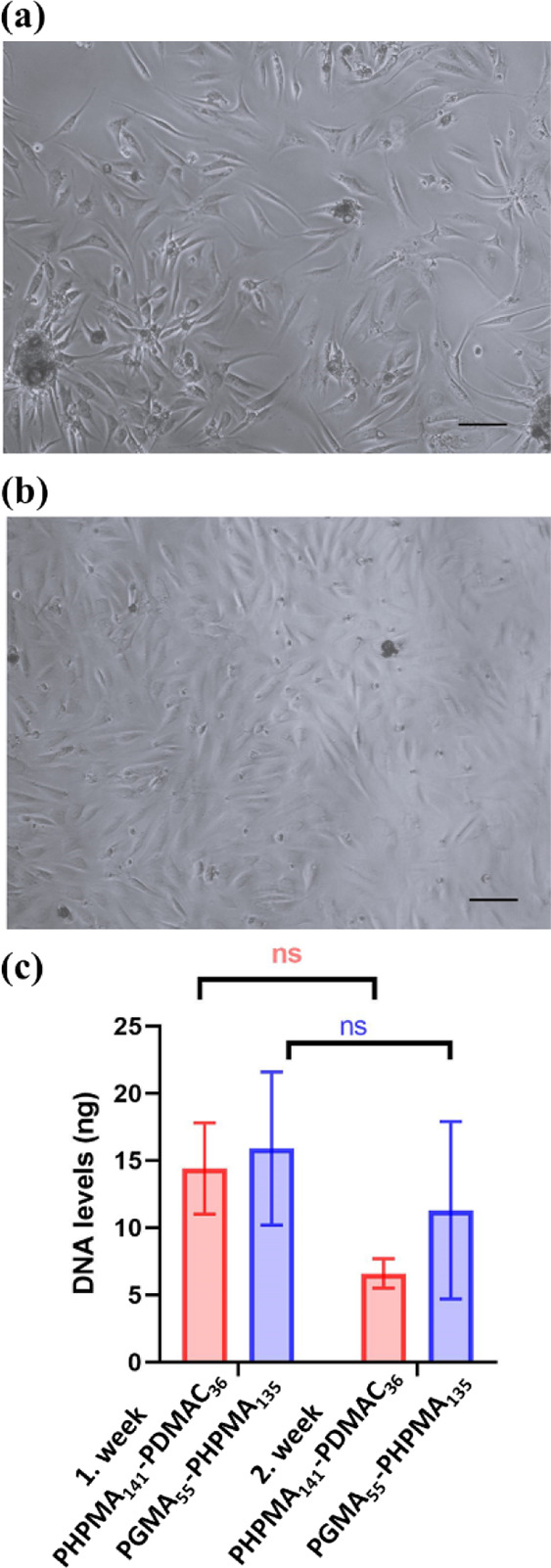
Phase contrast light microscopy images recorded for MSCs within 24 h of their release from (a) 4% w/w PHPMA_141_-PDMAC_36_ worm gel or (b) 6% w/w PGMA_55_-PHPMA_135_ worm gel after their encapsulation for 7 days at 37 °C. (c) Mass of DNA extracted from MSCs retrieved after 24 h of the monolayer culture following encapsulation within each worm gel for either 7 or 14 days at 37 °C. (Scale bars for both images are 100 μm; ns: nonsignificant, *P ≤ 0.01).

Figure 12a,b depicts the cell morphology observed within 24 h for MSCs released from worm gels. A reduction in cellular metabolic activity between day 14 and day 21 was also observed for MSCs encapsulated in the 4% w/w PHPMA_141_-PDMAC_36_ and 6% w/w PGMA_55_-PHPMA_135_ gels after their encapsulation for 7 days at 37 °C. In both cases, the released MSCs rapidly attached to the tissue culture plates and displayed a characteristic fibroblast-type morphology. Similar observations were made for MSCs after encapsulation for 14 days at 37 °C (images not shown).

MSCs proliferation or differentiation was assessed after their encapsulation within 150 μL aliquots of 4% w/w PHPMA_141_-PDMAC_36_ or 6% w/w PGMA_55_-PHPMA_135_ worm gels for either 7 or 14 days. In these experiments, MSC cells were retrieved from 3–4 individual 4% w/w PHPMA_141_-PDMAC_36_ or 6% w/w PGMA_55_-PHPMA_135_ gels at the desired time points by cooling to 4 °C to induce degelation, followed by centrifugation of the cold suspension to produce cell pellets. The resulting cells were resuspended in MSC culture media and then transferred to 24-well culture plates for 24 h to promote their attachment to the culture plates (Figure 12a,b).

The number of retrieved MSCs was quantified by determining the total DNA within these cells from each 150 μL gel using a Picogreen DNA assay standard curve for the Picogreen assay to which was used to determine the amount of DNA, see Figure S6 (performed in triplicate). The DNA levels were calculated using a typical standard curve for the Picogreen assay to which was used to determine the amount of DNA in the retrieved MSCs from the 150 μL 6% PHPMA_135_-PGMA_55_ gels or 150 μL 4% PHPMA_141_-PDMAC_39_ gels after 7 days, 14 days and 21 days. Fluorescence was read at excitation wavelength at 485 nm, and emission wavelength at 528 nm, gain = 85%. There was no significant difference between the total amounts of DNA extracted from MSCs retrieved after encapsulation within the two types of worm gels for either 7 or 14 days, see Figure 12c. However, lower levels of DNA were extracted from MSCs after incubation for 14 days within the 4% w/w PHPMA_141_-PDMAC_36_ worm gel. This became more significant (*P* ≤ 0.01) when MSCs were retrieved from the same gel after encapsulation for 21 days, see Figure S7. The uncertainty in such data reflects the differing number of viable cells retrieved from 150 μL aliquots. Nevertheless, this DNA assay confirms that there has been no significant cell proliferation during MSC encapsulation in either worm gel. This supports our central hypothesis that MSCs encapsulated within the 4% w/w PHPMA_141_-PDMAC_36_ worm gel enter stasis, as previously observed for pluripotent cells immersed within the 6% w/w PGMA_55_-PHPMA_135_ worm gel.^40^

Cell release from such thermoresponsive worm gels was readily achieved by using an ice bath to induce degelation. Such cold temperatures (4–5 °C) are routinely used for transportation of clinical samples. Degelation of the PHPMA_141_-PDMAC_36_ worm gel was somewhat slower than that of the PGMA_55_-PHPMA_135_ worm gel. This may be related to the lower number of cells retrieved from the former gel, which is supported by live-dead staining of freshly isolated retrieved cells (see Figure S8). The latter assay indicated that only 50% of MSCs retrieved from the 4% w/w PHPMA_141_-PDMAC_36_ worm gel were viable, whereas 85% viability was achieved when using the PGMA_55_-PHPMA_135_ worm gel (see Figure S7). The cell isolation protocol necessarily involves physical handling of the cells (e.g., pipetting, centrifugation, pelletization, resuspension, etc.). According to the literature, MSCs subjected to shear forces and/or vibration may be prone to apoptosis.^64^ Further optimization of the cell release protocol just below the critical gelation temperature and the use of a nonbicarbonate-buffered medium should enable the yield of viable MSCs to be improved.

**Figure 13:**
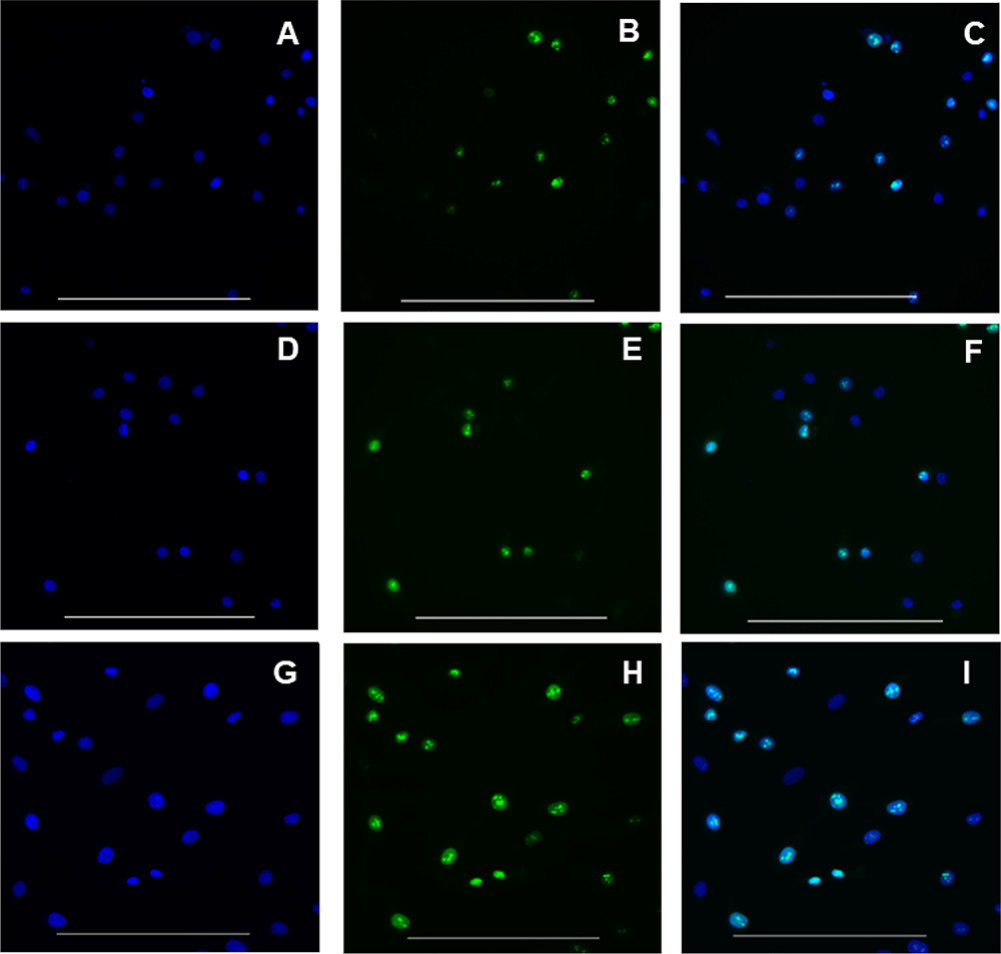
Immunocytochemical detection of the Ki-67 protein in MSCs retrieved from 4% w/w PHPMA_141_-PDMAC_36_ worm gel (images A–C), 6% w/w PGMA_55_-PHPMA_135_ worm gel (images D–F), and control MSCs cultured in monolayer culture (images G–I). MSCs were incubated with the Ki-67 antibody (green fluorescence) and nuclear stain DAPI (blue fluorescence). Images A, D, and G show DAPI fluorescence, while images B, E, and H show fluorescence arising from the Ki-67 antibody recorded for the same cells. Images C, F, and I show the merged images for A and B, D and E, and G and H, respectively. Scale bars for all images are 200 μm.

A Ki-67 assay was performed to examine whether MSCs were able to enter stasis when immersed within either worm gel, see Figure 13. The Ki-67 protein is not detected if cells are in their quiescent G_0_ state.^60^ In contrast, Ki-67 should be detected in all other stages of the cell cycle.

MSCs were retrieved from the 4% w/w PHPMA_141_-PDMAC_36_ (Figure 13A–C) and 6% w/w PGMA_55_-PHPMA_135_ worm gels (Figure 13D–F) after 14 days of encapsulation in the gels. After then, the MSCs were transferred to the monolayer culture for 24 h to enable cell attachment to the chamber slides prior to the immunocytochemistry assay. The Ki-67 protein was detected in 50% of MSCs within 24 h of their retrieval from the 4% w/w PHPMA_141_-PDMAC_36_ worm gel. This observation implies that 50% of retrieved MSCs had entered the cell cycle and were undergoing cell division. Additionally, this result also implies that the remaining 50% of the MSCs (i.e., those MSCs which did not express Ki-67 protein) were in stasis during the 14 day encapsulation period in the 4% w/w PHPMA_141_-PDMAC_36_ worm gel. Regarding the 6% w/w PGMA_55_-PHPMA_135_ worm gel, 43% of MSCs retrieved from the worm gel expressed the Ki-67 protein 24 h after retrieval thereby indicating these cells were entering the cell cycle to undergo proliferation. Hence, this result implies 57% of the retrieved MSCs did not express the Ki-67 protein and had entered stasis during the 14 days of their encapsulation in the 6% w/w PGMA_55_-PHPMA_135_ worm gel. The expression of Ki-67 protein by some of the MSCs may be a direct consequence of transferring the MSCs into the monolayer culture, which could stimulate the cells to enter the cell cycle.

However, the proportion of Ki-67-stained MSCs was significantly higher (70%) for actively proliferating monolayer cultures, see Figure 13H.

Thus, for the monolayer MSC cultures, only 30% of cells were quiescent at the time of the assay. Unfortunately, it is not possible to identify the precise stage of the cell cycle for the Ki-67-positive MSCs because Ki-67 is produced in all stages except G_0_.^60^ After monolayer culture for 24 h after cell retrieval, 50% of MSCs had not entered the quiescent G_0_ stage of the cell cycle. In principle, placing the retrieved MSCs in a monolayer culture environment could initiate their re-entry into the cell cycle. The control assay confirmed that the secondary antibody did not produce nonspecific immunofluorescence in the absence of the Ki-67 primary antibody (see Figure S9). However, we could not determine whether these MSCs had already entered the cell cycle before their retrieval from the worm gels or whether the retrieval process and/or monolayer conditions had initiated this effect.

Encapsulation of MSCs within 4% w/w PHPMA_141_-PDMAC_36_ and 6% w/w PGMA_55_-PHPMA_135_ worm gels for up to two weeks at 37 °C did not appear to affect their capacity to undergo differentiation after retrieval. The isolated MSCs were subjected to a further passage as a monolayer culture prior to incubation either in an osteogenic differentiation medium (to stimulate osteoblastic differentiation) or a basal medium (to detect basal levels of spontaneous differentiation).

**Figure 14:**
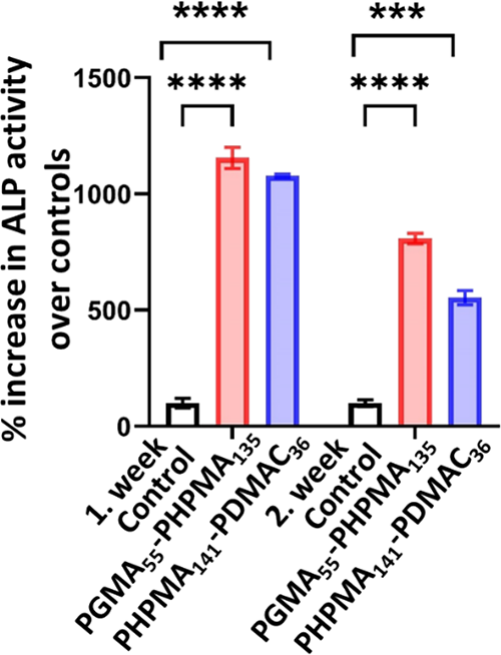
Osteogenic differentiation capacity of MSCs retrieved after encapsulation for either one or two weeks within the 4% w/w PHPMA_141_-PDMAC_36_ or 6% w/w PHPMA_141_-PDMAC_36_ worm gel. The retrieved MSCs were incubated in either a control or osteogenic differentiation medium followed by determination of the alkaline phosphatase enzyme activity. The retrieved MSCs exhibit high levels of alkaline phosphatase activity. In contrast, the control cells do not express high levels of alkaline phosphatase activity (****P ≤ 0.0001).

Alkaline phosphatase is a reliable marker of osteoblastic differentiation.^57,71^ Incubation of the retrieved MSCs in a standard osteogenic differentiation media yielded cells with significantly higher alkaline phosphatase activity (*P* ≤ 0.0001, Figure 14), regardless of whether they had been encapsulated within PHPMA_141_-PDMAC_36_ or PHPMA_135_-PGMA_55_. In contrast, the control cells that had not been exposed to the osteogenic differentiation medium expressed very low levels of alkaline phosphatase. This indicates that neither worm gel induces osteogenic differentiation.

MSC cells remained viable after encapsulation within each worm gel for at least two weeks at 37 °C. Thus, both the 4% w/w PHPMA_141_-PDMAC_36_ and 6% w/w PHPMA_135_ PGMA_55_ worm gels are biocompatible. Cell metabolic activity remained low for MSCs placed within the PHPMA_135_-PGMA_55_ worm gel but some increase in metabolic activity was observed for MSCs encapsulated within the PHPMA_141_-PDMAC_36_ worm gel. This suggests that the extent of cell proliferation within the gels was very low compared to that of monolayer cultures, for which the MSCs have a population doubling time of two days.^68^

Moreover, after encapsulation, thermally induced degelation aids cell retrieval and yields MSCs that exhibit excellent capacity for proliferation while maintaining their essential “stemness” during worm gel encapsulation.

In 2016, we reported that PGMA_55_-PHPMA_135_ worm gels can be used to induce stasis in human pluripotent stem cells (hPSC).^40^ In a follow-up study, we found that the hydroxyl-rich nature of the PGMA chains seemed to be important for stasis induction: if this steric stabilizer is replaced with poly(ethylene oxide), then cell proliferation occurs within a PEG_57_-PHPMA_65_ worm gel.^39^ In the present study, proliferation appears to be suppressed for MSCs encapsulated within a PHPMA_141_-PDMAC_36_ worm gel, although perhaps not to quite the same extent as that observed for the PGMA_55_-PHPMA_135_ worm gel. Nevertheless, DMAC monomer is much cheaper than the GMA monomer, so the former gel could prove to be useful for the long-term storage or global transportation of live MSCs for regenerative medicine applications and perhaps also for bone marrow transplantation.

## Conclusions

In summary, we have prepared a series of new PHPMA_*x*_-PDMAC_*y*_ diblock copolymers via RAFT solution polymerization. GPC studies confirmed low dispersities, and ^1^H NMR spectroscopy studies indicate high conversions for the second-stage DMAC polymerization. Importantly, our prior experience of the synthesis of similar amphiphilic diblock copolymers via aqueous PISA—plus the construction of an appropriate pseudo-phase diagram—has provided useful guidelines for targeting suitable diblock copolymer compositions to produce soft, free-standing PHPMA_141_-PDMAC_36_ worm gels in semiconcentrated aqueous solution via initial dissolution in cold water. If desired, well-defined spheres and vesicles can also be targeted for this diblock copolymer system by adjusting the target PDMAC DP accordingly.

PHPMA_141_-PDMAC_36_ worm gels undergo reversible degelation on cooling from 37 to 4 °C owing to a worm-to-sphere transition, as confirmed by TEM analysis and rheology studies. Variable temperature ^1^H NMR studies conducted between 5 and 50 °C confirmed a higher degree of hydration for the weakly hydrophobic PHPMA block at lower temperature, which is consistent with the observed morphological transition. It is perhaps worth emphasizing that the PDMAC block facilitates such studies because its proton signals do not overlap with those assigned to the PHPMA (unlike alternative hydrophilic blocks). Hence the present study provides more compelling NMR evidence for the thermoresponsive nature of the weakly hydrophobic PHPMA block compared to that reported in the literature.^30,38^

The rheological behavior of the PHPMA_141_-PDMAC_36_ worm gel is remarkably similar to that previously reported for PEG_57_-PHPMA_65_ and PGMA_55_-PHPMA_135_ worm gels.^39,40^ Thus we examined whether such non-ionic PDMAC chains promote stasis induction (like PGMA) or cell proliferation (like PEG chains). Cell biology studies confirmed that human bone marrow MSCs remained viable when encapsulated for two weeks within either 4% w/w PHPMA_141_-PDMAC_36_ or 6% w/w PGMA_55_-PHPMA_135_ worm gels. In each case, cell viability and DNA assays indicated that minimal MSC proliferation occurred after two weeks encapsulation at 37 °C. However, approximately 50% MSCs expressed the well-known cell proliferation marker, Ki-67, during their encapsulation within the 4% w/w PHPMA_141_-PDMAC_36_ worm gel. Nevertheless, there was no significant increase in the total cell number (as determined by the total amount of DNA). For comparison, approximately 43% MSCs expressed Ki-67 when encapsulated within a 6% w/w PGMA_55_-PHPMA_135_ worm gel over the same time period. It is feasible that MSC retrieval from the worm gels and/or their subsequent transfer to monolayer culture initiated their re-entry into the cell cycle.

According to an alkaline phosphatase marker used to examine their osteoblastic differentiation capacity, MSCs exhibited a significant increase in ALP activity after encapsulation for 1–2 weeks within either a 4% w/w PHPMA_141_-PDMAC_36_ or a 6% w/w PGMA_55_-PHPMA_135_ worm gel. Hence MSCs retain their capacity for stimulated differentiation after retrieval from either worm gel.

Overall, the PDMAC_39_ steric stabilizer block induced similar cell behavior to that of the hydroxyl-rich PGMA steric stabilizer previously reported by us.^39,40^ In principle, the worm gels described herein could be used for the storage or global transportation of live MSCs for up to two weeks. This may be sufficient for regenerative medicine applications and perhaps also for bone marrow transplantation. In this context, it is worth emphasizing that DMAC monomer is much more cost-effective than GMA.

## Supporting Information Available

^1^H NMR spectra recorded for MePETTC RAFT agent and the PHPMA_141_ precursor, additional oscillatory rheology data obtained for PHPMA_148_-PDMAC_39_ worms as a function of copolymer concentration and temperature in the absence and presence of PBS, schematic synthesis of PHPMA_141_-PDMAC_36_-SH diblock copolymer from a PHPMA_141_-PDMAC_36_-S-S-PDMAC_36_-PHPMA_141_ triblock copolymer, digital photographs of freeze-dried PHPMA_141_-PDMAC_36_ diblock copolymer powder recorded before and after end-group removal, ^1^H NMR spectra to confirm successful RAFT end-group removal for the PHPMA_141_-PDMAC_36_ diblock copolymer, linear calibration curve for the Picogreen assay used to determine the mass of DNA after MSC encapsulation, mass of DNA recorded for MSCs retrieved from two worm gels after encapsulation for 21 days at 37 °C, fluorescence microscopy images recorded for a live/dead assay, and fluorescence microscopy control images obtained for the immunocytochemical detection of Ki-67 (PDF)
