# Supplementary material for: Thermoresponsive Poly(N,N′-dimethylacrylamide)-Based Diblock Copolymer Worm Gels via RAFT Solution Polymerization: Synthesis, Characterization, and Cell Biology Applications
Source: Biomacromolecules. 2023 Aug 24;24(9):4285–302. doi: 10.1021/acs.biomac.3c00635 (PMC10498450; doi:10.1021/acs.biomac.3c00635)
Supplement: Supplementary file 1 — bm3c00635_si_001.pdf [file bm3c00635_si_001.pdf]

Supporting Information for:

Thermoresponsive Poly(N,N'-dimethylacrylamide)-based Diblock Copolymer Worm Gels via RAFT Solution Polymerization: Synthesis, Characterization and Cell Biology Applications

Damla Ulker,[1,2]* Thomas J. Neal,[1] Aileen Crawford[3] and Steven P. Armes[1]*

1. *Dainton Building, Department of Chemistry, University of Sheffield, Brook Hill, Sheffield, S3 7HF, South Yorkshire, UK.*
2. *Faculty of Pharmacy, Department of Pharmaceutical Basic Sciences, Near East University, Nicosia, TR-99138, Northern Cyprus, Turkey.*
3. *School of Clinical Dentistry, University of Sheffield, Claremont Crescent, Sheffield, S10 2TA, South Yorkshire, UK.*

Summary of Contents

Figure S1. ^{1}H NMR spectra recorded for the MePETTC RAFT agent and the $PHPMA_{141}$ precursor.

Figure S2. Oscillatory rheology data obtained for $PHPMA_{148}$-$PDMAC_{39}$ worms of varying copolymer concentration at (a) 20 °C and (b) 37 °C. Oscillatory rheology data obtained for a 10% w/w aqueous dispersion of $PHPMA_{148}$-$PDMAC_{39}$ worms as a function of temperature in (c) the absence and (d) the presence of PBS.

Figure S3. Schematic synthesis of a $PHPMA_{141}$-$PDMAC_{36}$-SH diblock copolymer by cleavage of the disulfide bond within a $PHPMA_{141}$-$PDMAC_{36}$-S-S-$PDMAC_{36}$-$PHPMA_{141}$ triblock copolymer.

Figure S4. Digital images of freeze-dried $PHPMA_{141}$-$PDMAC_{36}$ diblock copolymer powder recorded before and after end-group removal.

Figure S5. ^{1}H NMR spectra to confirm successful RAFT end-group removal for the $PHPMA_{141}$-$PDMAC_{36}$ diblock copolymer.

Figure S6. Linear calibration curve obtained for the Picogreen assay used to determine the mass of DNA after MSC encapsulation within worm gels.

Figure S7. Mass of DNA recorded for MSCs retrieved from two worm gels after encapsulation for 21 days at 37 °C.

Figure S8. Fluorescence microscopy images obtained for a live/dead assay.

Figure S9. Fluorescence microscopy control images obtained for the immunocytochemical detection of Ki-67.

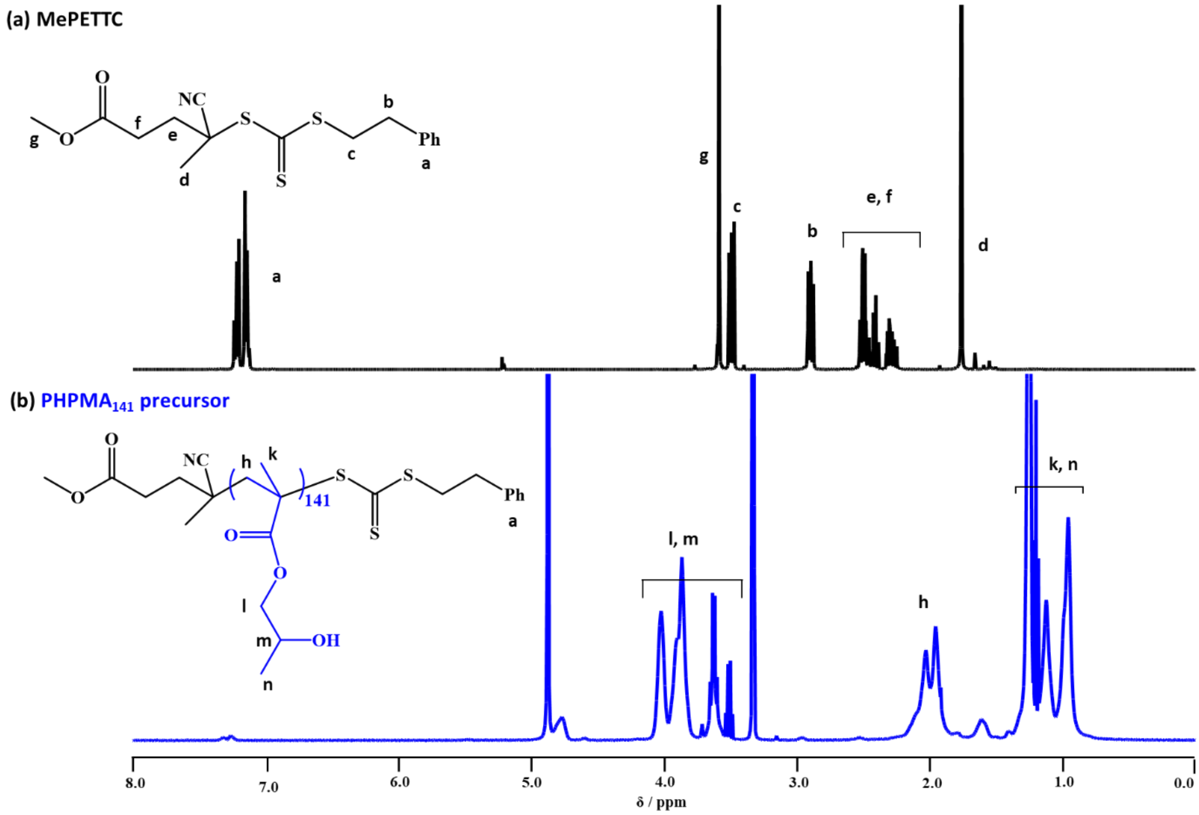

Figure S1. ^{1}H NMR spectra (CD_3OD) recorded for (a) the MePETTC RAFT agent and (b) a $PHPMA_{141}$ precursor.

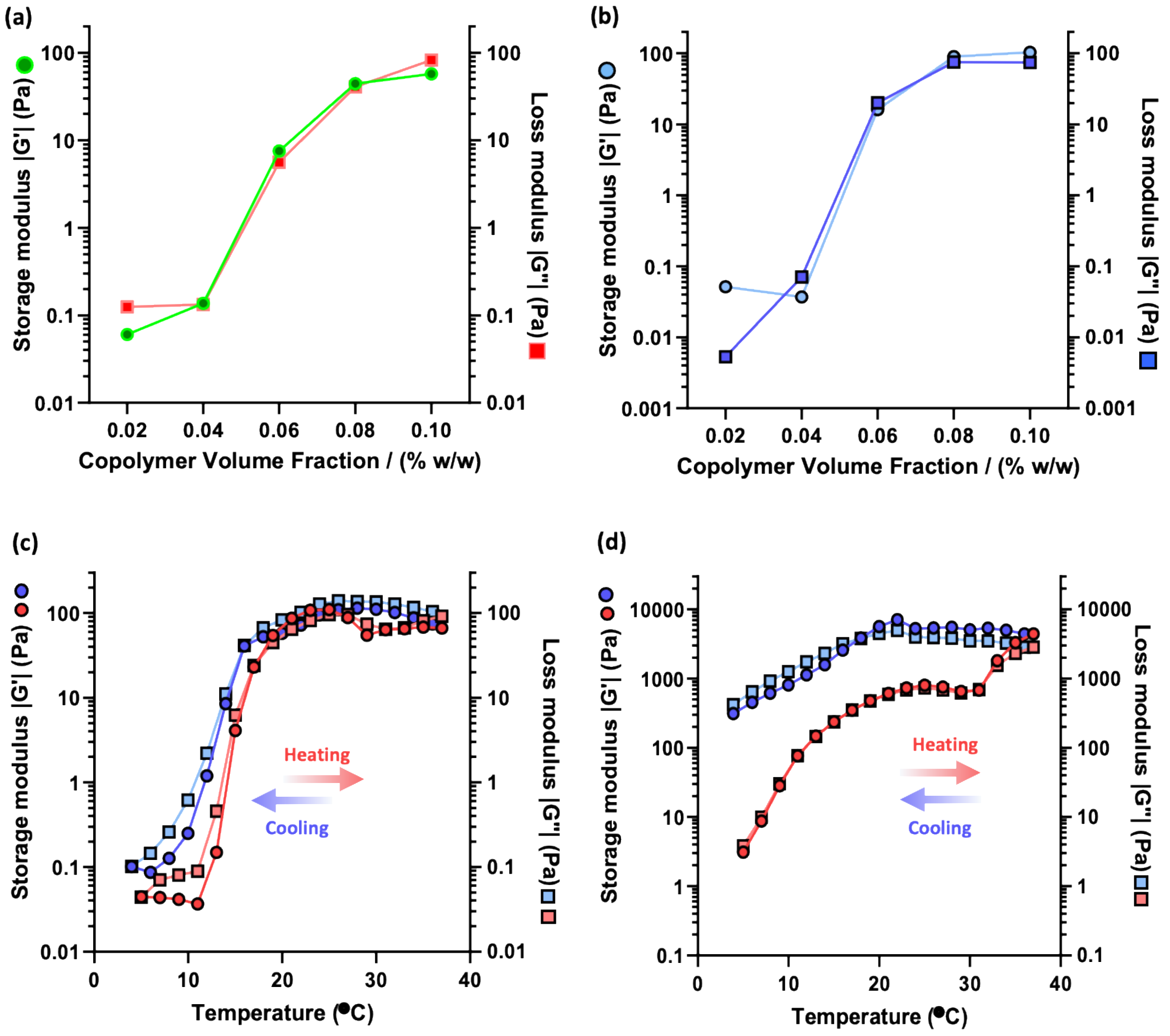

Figure S2. Oscillatory rheology data obtained (a) at 20 °C and (b) at 37 °C for a series of PHPMA$_{148}$-PDMAC$_{39}$ worms of varying copolymer concentration. [N.B. G' data are denoted by solid circles (green for 20 °C and light blue for 37 °C) and G'' data are denoted by solid squares (red for 20 °C and blue for 37 °C)]. (c) Variable temperature oscillatory data obtained during heating (G' data denoted by red solid circles, and G'' data denoted by light red solid squares) and cooling (G' data denoted by blue solid circles, and G'' data denoted by light blue solid squares) for a 10% w/w aqueous dispersion of PHPMA$_{148}$-PDMAC$_{39}$ worms. (d) Variable temperature oscillatory data obtained during heating (G' data denoted by red solid circles, and G'' data denoted by light red solid squares) and cooling (G' data denoted by blue solid circles, and G'' data denoted by light blue solid squares) for a 10% w/w aqueous dispersion of PHPMA$_{148}$-PDMAC$_{39}$ worms in the presence of PBS. All oscillatory rheology experiments were conducted at a strain of 1.0% and an angular frequency of 1.0 rad s^{-1}.

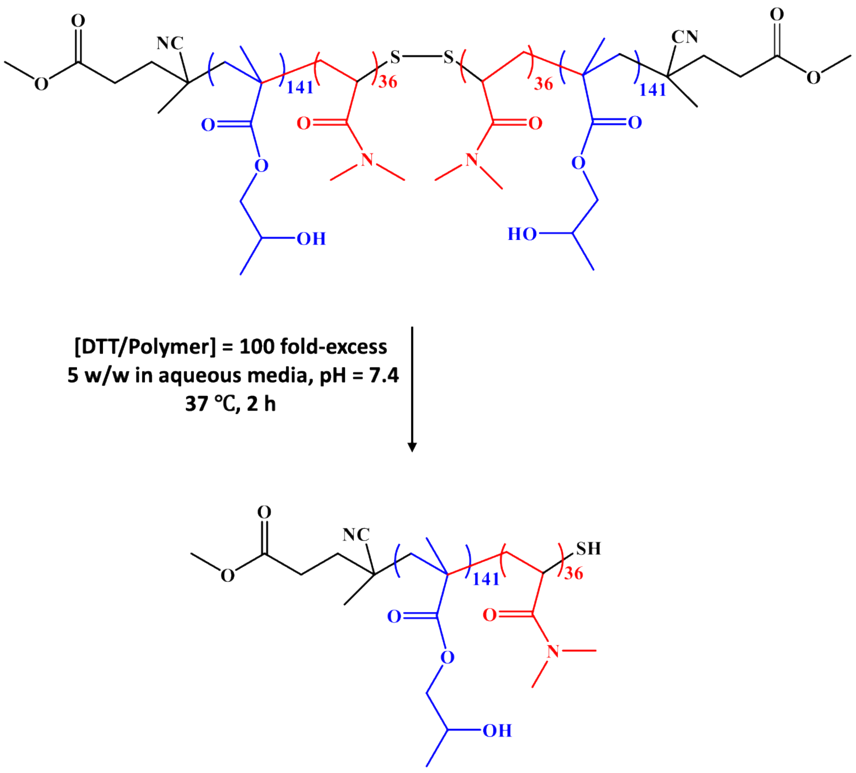

Figure S3. Schematic representation of the cleavage of the disulfide bond within a $PHPMA_{141}$-$PDMAC_{36}$-S-S-$PDMAC_{36}$-$PHPMA_{141}$ triblock copolymer to yield a $PHPMA_{141}$-$PDMAC_{36}$-SH diblock copolymer. Conditions: 5.0% w/w copolymer in aqueous media; DTT/copolymer molar ratio = 100; pH 7.4.

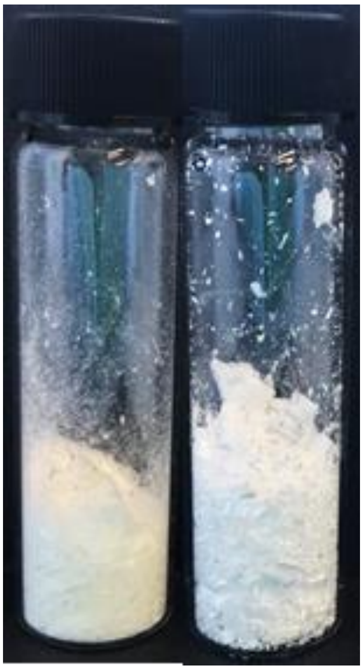

Figure S4. Digital photographs recorded for freeze-dried $PHPMA_{141}$-$PDMAC_{36}$ diblock copolymer powder before and after end-group removal. Note the change in color from pale yellow (left vial) to white (right vial).

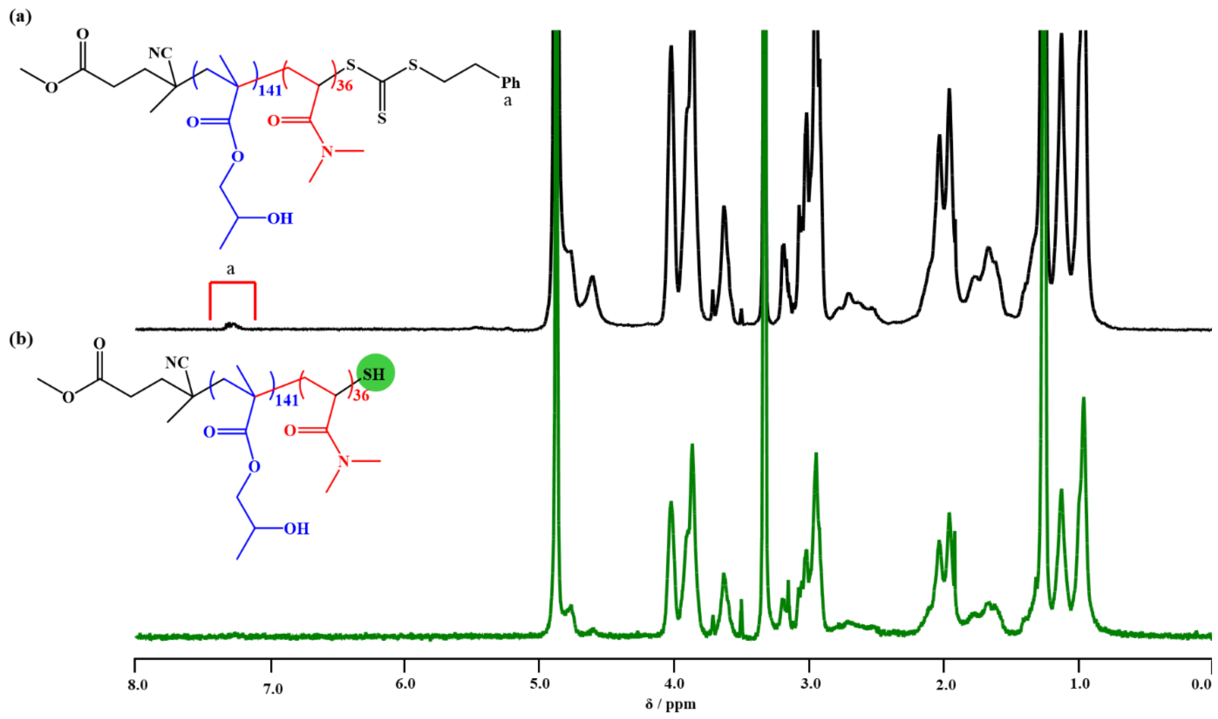

Figure S5. ^{1}H NMR spectra (CD_3OD) recorded (a) an as-synthesized $PHPMA_{141}$-$PDMAC_{36}$ diblock copolymer and (b) the same copolymer after removal of its RAFT end-groups by using a twenty-fold excess of propylamine at 20 ℃.

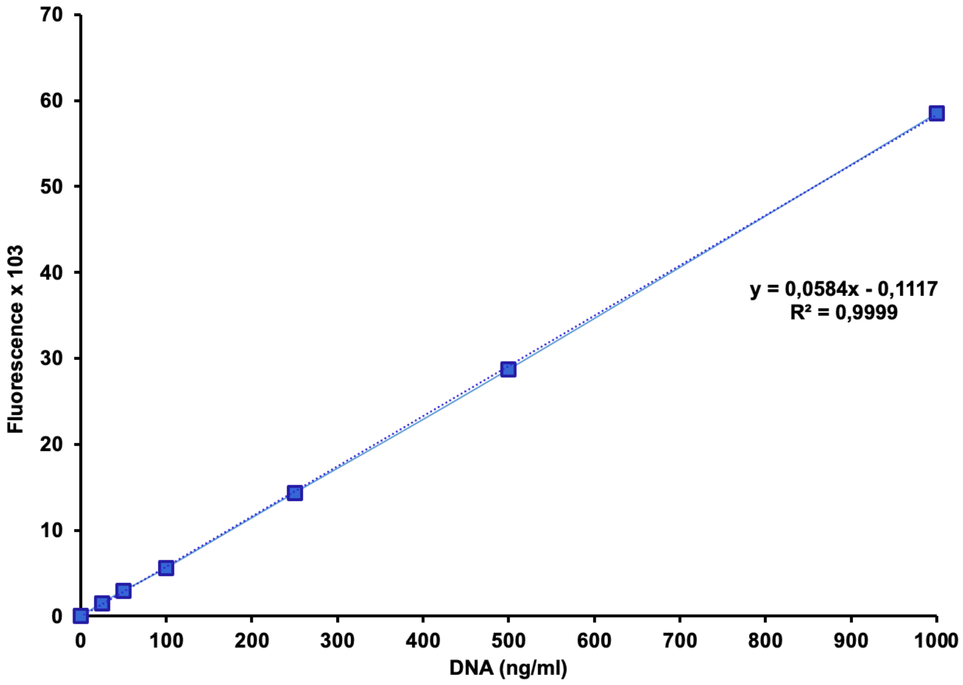

Figure S6. Linear calibration curve obtained for the Picogreen assay used to determine the amount of DNA in MSCs retrieved from 6% $PHPMA_{135}$-$PGMA_{55}$ gels or 4% $PHPMA_{141}$-$PDMAC_{39}$ gels. Fluorescence was recorded at 85% gain using an excitation wavelength of 485 nm and an emission wavelength of 528 nm.

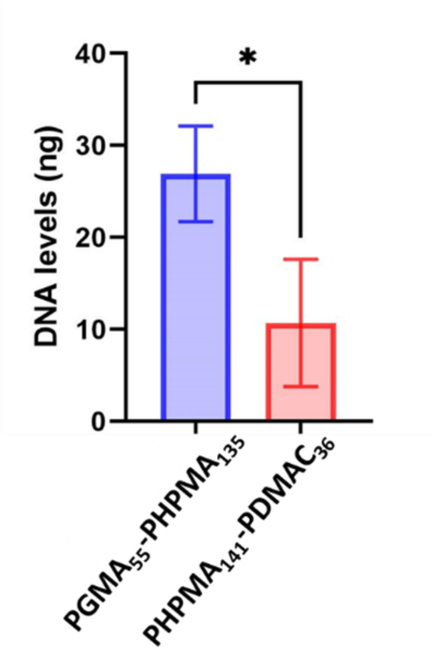

Figure S7. Mass of DNA retrieved from MSCs after their encapsulation for 21 days at 37 °C within either 4% w/w $PHPMA_{141}$-$PDMAC_{36}$ worm gel or 6% w/w $PGMA_{55}$-$PHPMA_{135}$ worm gel. (*P ≤ 0.01).

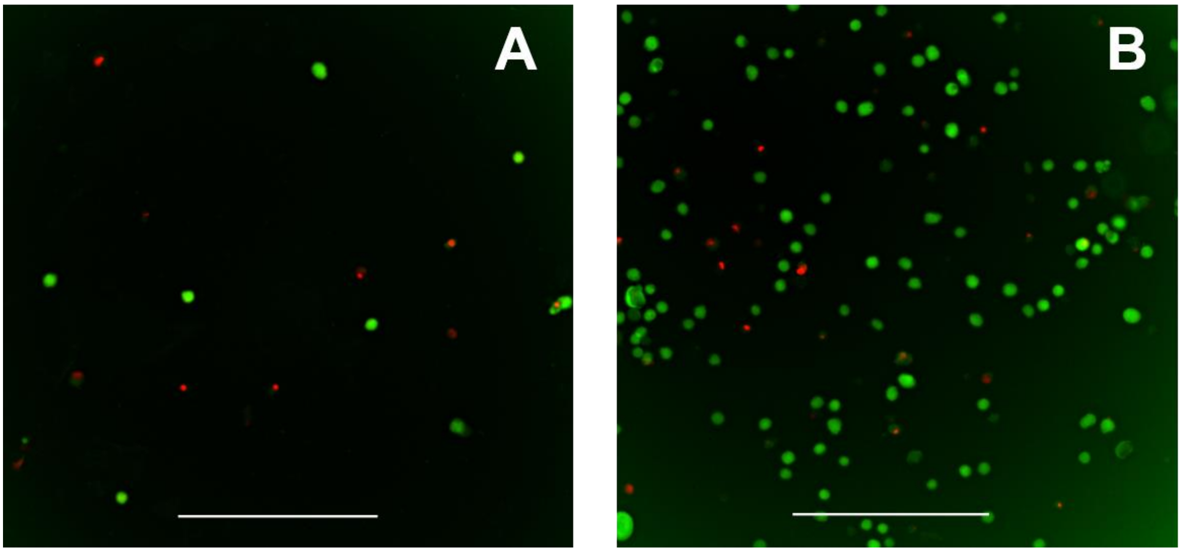

Figure S8. Fluorescence microscopy images recorded for live/dead staining of MSCs retrieved after 14 days encapsulation within a 4% w/w $PHPMA_{141}$-$PDMAC_{36}$ worm gel (image A) or a 6% w/w $PHPMA_{135}$-$PGMA_{55}$ worm gel (image B). Green fluorescence indicates uptake of the CMFDA (live stain) within the cytoplasm of viable MSCs. Red fluorescence indicates the uptake of propidium iodide (dead stain) within the nuclei of dead/dying MSCs. Scale bar = 100 µm in each case.

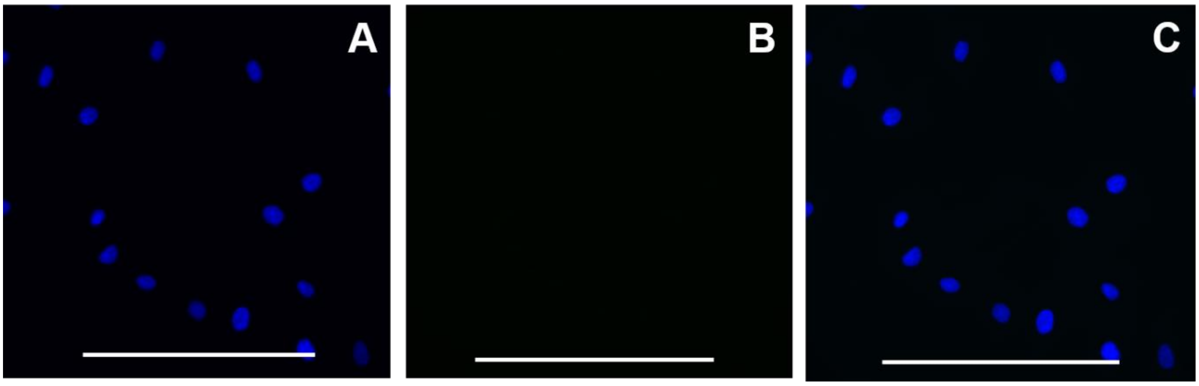

Figure S9. Fluorescence microscopy images recorded for the immunocytochemical detection of Ki-67. In this control experiment, the staining protocol was performed for actively proliferating monolayer cultures of MSCs in the absence of the primary antibody to Ki-67. Image A shows the blue fluorescence from the nuclear stain, DAPI. Image B confirms that no green fluorescence is observed with the secondary antibody when the primary (non-fluorescent) antibody to Ki67 is omitted. This lack of fluorescence demonstrates that the binding between the Ki67 antibody and the secondary fluorescent antibody is specific - there is no non-specific binding with the secondary fluorescent antibody. Image C is a merged image combining image A and image B. Scale bar = 200 µm in each case.
